# The Core Apoptotic Executioner Proteins CED-3 and CED-4 Promote Initiation of Neuronal Regeneration in *Caenorhabditis elegans*

**DOI:** 10.1371/journal.pbio.1001331

**Published:** 2012-05-22

**Authors:** Berangere Pinan-Lucarre, Christopher V. Gabel, Christopher P. Reina, S. Elizabeth Hulme, Sergey S. Shevkoplyas, R. Daniel Slone, Jian Xue, Yujie Qiao, Sarah Weisberg, Kevin Roodhouse, Lin Sun, George M. Whitesides, Aravinthan Samuel, Monica Driscoll

**Affiliations:** 1 Department of Molecular Biology and Biochemistry, Rutgers University, Piscataway, New Jersey, United States of America; 2 Department of Physics and Center for Brain Science, Harvard University, Cambridge, Massachusetts, United States of America; 3 Department of Chemistry and Chemical Biology, Harvard University, Cambridge, Massachusetts, United States of America; 4 Department of Biomedical Engineering, Tulane University, New Orleans, Louisiana, United States of America; 5 Department of Physiology and Biophysics, Boston University School of Medicine, Boston, Massachusetts, United States of America; The Rockefeller University, United States of America

## Abstract

A critical accomplishment in the rapidly developing field of regenerative medicine will be the ability to foster repair of neurons severed by injury, disease, or microsurgery. In *C. elegans*, individual visualized axons can be laser-cut in vivo and neuronal responses to damage can be monitored to decipher genetic requirements for regeneration. With an initial interest in how local environments manage cellular debris, we performed femtosecond laser axotomies in genetic backgrounds lacking cell death gene activities. Unexpectedly, we found that the CED-3 caspase, well known as the core apoptotic cell death executioner, acts in early responses to neuronal injury to promote rapid regeneration of dissociated axons. In *ced-3* mutants, initial regenerative outgrowth dynamics are impaired and axon repair through reconnection of the two dissociated ends is delayed. The CED-3 activator, CED-4/Apaf-1, similarly promotes regeneration, but the upstream regulators of apoptosis CED-9/Bcl2 and BH3-domain proteins EGL-1 and CED-13 are not essential. Thus, a novel regulatory mechanism must be utilized to activate core apoptotic proteins for neuronal repair. Since calcium plays a conserved modulatory role in regeneration, we hypothesized calcium might play a critical regulatory role in the CED-3/CED-4 repair pathway. We used the calcium reporter cameleon to track in vivo calcium fluxes in the axotomized neuron. We show that when the endoplasmic reticulum calcium-storing chaperone calreticulin, CRT-1, is deleted, both calcium dynamics and initial regenerative outgrowth are impaired. Genetic data suggest that CED-3, CED-4, and CRT-1 act in the same pathway to promote early events in regeneration and that CED-3 might act downstream of CRT-1, but upstream of the conserved DLK-1 kinase implicated in regeneration across species. This study documents reconstructive roles for proteins known to orchestrate apoptotic death and links previously unconnected observations in the vertebrate literature to suggest a similar pathway may be conserved in higher organisms.

## Abstract 2

Laser severing of individual axons in the nematode *Caenorhabditis elegans* revealed that the apoptotic executioner caspase CED-3 and its regulator CED-4/Apaf-1 play an unexpected beneficial role in promoting axonal regeneration.

## Author Summary

Clinical success in reconnecting neurons damaged by injury will require detailed molecular understanding of how mature axons respond to being severed. To decipher intrinsic molecular pathways that stimulate axon regeneration, we use the small transparent model, *Caenorhabditis elegans*, in which individual labeled axons can be laser-severed without damage to neighboring tissue, and regrowing axons can be observed directly in the living animal. We find that the apoptotic protein CED-3, well known for its developmental roles in cell death, also unexpectedly acts in a beneficial role to promote regeneration of severed axons. Initial post-surgery outgrowth is impaired in a *ced-3* mutant, suggesting that CED-3 is involved in the early steps of axonal regeneration. The activation of CED-3 caspase in this context occurs independently of major cell death regulatory pathways, but efficient regeneration does require the caspase activator CED-4/Apaf-1, the conserved regeneration kinase DLK-1, and calreticulin-dependent calcium fluxes within the severed neuron. Our data suggest a novel conserved pathway for neuronal reconstruction, and call into question the practice of blocking caspases to treat neuronal injury in the clinic.

## Introduction

In the injured vertebrate central nervous system (CNS), neurons often survive and sprout but encounter extrinsic and intrinsic barriers to functional regeneration [1], with devastating consequences for victims. The successful repair of neurons severed by accident or surgery is an obvious goal of modern regenerative medicine. A more detailed understanding of the fundamental molecular mechanisms of neuronal regeneration within a physiological context will be required for design of novel and effective therapies that could shift treatment goals from palliative care to restoration of function.

Considerable understanding of regeneration responses consequent to neuronal injury has been generated via study of vertebrate models in vivo and in vitro. More recently, laser technology advanced the precision of in vivo investigation to the single axon level by enabling the axotomy of individual processes in genetic model organisms [2],[3]. Moreover, the opportunity to test individual gene activities for roles in regeneration biology in whole animal context, and now to conduct high throughput genetic and pharmacological screens for such activities [4]–[6], is contributing to rapid advances in dissection of molecular mechanisms involved in neuronal regeneration. Although very much a work in progress, the emerging picture suggests regeneration may employ mechanisms conserved across species [5]. For example, in *Caenorhabditis elegans*, like in other models, physical disruption of an axon triggers an intracellular calcium spike [7],[8]. Calcium waves can originate from extracellular sources via voltage-gated calcium channels and may be amplified by release from internal stores. Elevation of calcium concentration activates signaling pathways, notably cAMP and MAPK DLK-1 pathways [8]–[10], which control growth cone formation and subsequent axonal elongation through cytoskeleton and membrane remodeling. Many details of the complex mechanisms involved remain to be established, the accomplishment of which might inspire strategies for directed neuronal repair.

With an initial interest in whether neurons might activate death pathways to eliminate the dissociated fragments generated by axon severing, we performed femtosecond laser microsurgeries on individual *C. elegans* neurons that lacked cell death proteins. To our surprise, we found that dissociated fragments often persisted for significant amounts of time. Moreover, CED-3 caspase, the essential core executioner protease in apoptosis [11], rather than being needed for cell fragment elimination, instead acts beneficially to promote early events in neuronal regeneration. *ced-3* mutations affect early regenerative dynamics with the consequence of slowing initial outgrowth and delaying the physical reconnection of the regenerating axon to the severed distal segment, although *ced-3* deficiency does not change long-term regeneration outcome. Core apoptotic proteins CED-3 and CED-4 are mobilized via a regulatory mechanism distinct from that involving known apoptotic regulators but which requires calcium flux and regeneration kinase *dlk-1*. Our data pull together disconnected observations in the literature to suggest that caspases act via a conserved mechanism to promote regenerative responses in injured neurons.

## Results

### CED-3 Caspase Activity Is Needed for Efficient Axonal Regeneration

**Figure 1:**
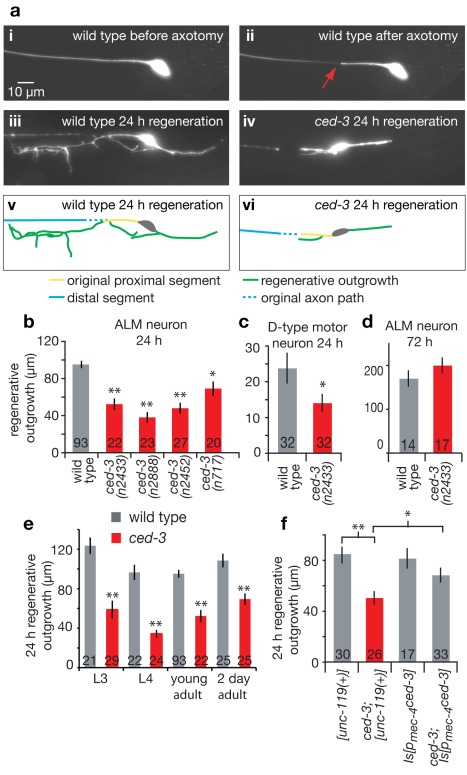
CED-3 caspase activity is needed for efficient axonal regeneration. (a) Representative images of a *p_mec-4_*GFP-labeled ALM neuron were taken before (i), immediately after (ii, red arrow indicates cut point at 20 µm from the cell body), and 24 h after laser axotomy in WT (iii) and in the *ced-3(n2433)* active site mutant (iv). Images are projected z-stacks. The green traces indicate the observed regenerative outgrowth for WT (v) and *ced-3(n2433)* (vi); scale bar: 10 µm. Regenerative outgrowth was measured 24 h after surgery in (b) ALM neurons (four independent *ced-3* alleles including deletion allele *n2452* and active site mutant allele *n2433*) and (c) D-motor neurons for young adult animals. (d) Regenerative outgrowth was measured 3 d after surgery in ALM neurons in WT and *ced-3(n2433)* (no statistical difference between the two by *t* test). (e) Comparison of WT (grey) and *ced-3(n2433)* (red) 24 h regenerative outgrowth in ALM neurons for different age animals (L3 and L4 larvae, young adults, and 2-d-old adult). (f) Cell autonomy test for *ced-3* rescue of regeneration outgrowth phenotype. The length of ALM regenerative outgrowth was measured 24 h after surgery in young adult animals for the control transgenic strains, bearing the *unc-119(+)* marker of transformation, *Is[unc-119(+)]* and *ced-3(n2433); Is[unc-119(+)]* as well as transgenic strains expressing *ced-3* in the touch neurons *Is[p_mec-4_ced-3]* and *ced-3(n2433); Is[p_mec-4_ced-3]*. See notes on strain construction and *ced-3* transgene expression toxicity in touch neurons in Figure S2. The *unc-119* integrated copy (*Is[unc-119(+)]*) did not affect the *ced-3(n2433)* defect in regeneration, and expression of *p_mec-4_ced-3* (*Is[p_mec-4_ced-3]*) in the mechanosensory neurons rescues the *ced-3(n2433)* defect, despite some neurotoxicity. *p_mec-4_ced-3* expression in wild type does not induce excessive regeneration (panel f, third bar), and thus does not appear sufficient to promote regeneration, although we cannot rule that toxicity of elevated caspase activation could mask a potential beneficial outcome. All bar graphs depict mean ± s.e.m. The Student's *t* test, with a Dunn-Sidak adjustment for multiple comparisons, was used to determine the statistical significance of differences versus WT in each panel, except in (f) where brackets indicate direct Student's *t* test between two specific values; *p<0.05, **p<0.005 in all cases. Number of animals assayed is indicated in (or above) each bar for this and all other figures.

With an initial interest in whether neurons might activate death pathways in soma or dissociated fragments in response to severe physical injury such as axon severing, we performed femtosecond laser microsurgeries on individual GFP-visualized *C. elegans* neurons. We find that ALM mechanosensory neurons and D-type motor neurons rarely die after laser axotomy in adult *C. elegans*. Moreover, the severed dissociated processes generally persist for several days post-surgery (Figure S1) and can remain functional, as axotomized animals were touch-sensitive 6 h after surgery and remained so up to at least 1 wk post-surgery (see data note in Materials and Methods). As observed previously [2],[3],[9],[12],[13], severed processes display substantial regeneration from the soma-proximal side, with the severed stump regenerating a structure that first extends multiple spike-like filopodia and then directs further axonal extension (see Movie S1 for a typical depiction of wild type (WT) regeneration). At 24 h, roughly one third of axotomized ALM axons grew back to track along the severed distal process (see below), and the remaining axons displayed dramatic outgrowth with long and branched processes (Figure 1ai–iii, v). We also noted limited regrowth responses from the end of the severed soma-distal side (see below and Movies S1 and S2).

As one approach toward quantitation of the regeneration response, we measured total new outgrowth length of the proximal fragment 24 h following laser surgery for those neurons that did not regrow back into the original severed process (Figure 1aiii, v; those processes that did track back to the old distal process could not be measured as the new process could not be distinguished from the old persisting process). Somewhat unexpectedly, four independent mutants of *ced-3* caspase, the central apoptosis executor protease required for all *C. elegans* programmed cell deaths [11], showed markedly reduced regenerative ALM outgrowth in this timeframe (Figure 1aiv, vi, 1b), a phenotype also exhibited by *ced-3* D-type motor neurons (Figure 1c). *ced-3* regenerative defects in severed ALM neurons diminished with time and were no longer apparent at 3 d post-surgery (Figure 1d). Most severe ALM deficits in *ced-3* mutants occur in L4 larvae, although significant regeneration differences are apparent in young adults (Figure 1e). Notably, mutant phenotypes in the *ced-3(n2433)* active-site point mutant, which is deficient in in vitro protease activity [14], indicate that caspase activity itself is necessary for efficient axonal regeneration.

Axonal regeneration involves a complex interplay of biochemical activities within the injured neuron and interactions of the neuron with signals and structures in its environment. Thus, caspases might act directly in injured neurons, in the synaptic partners that provide guidance cues, or in the surrounding tissue (hypodermis for touch neurons) to set up conditions permissive for regeneration. To address whether CED-3 caspase activity is required within the severed neuron to facilitate regeneration, we expressed *ced-3* in the mechanosensory neurons of the *ced-3(n2433)* mutant. Although like others [15] we found that expression of caspase transgenes is most often associated with cell toxicity, making the generation of transgenic lines extremely challenging, we identified one low copy number transgenic line with only moderate touch neuron loss (Figure S2). We found that the regeneration defect induced by *ced-3(n2433)* was rescued by specific expression of *ced-3* in the mechanosensory neurons (Figure 1f), supporting that CED-3 acts in the damaged neuron for regeneration. Of note, moderate overexpression of *ced-3* in wild-type neurons did not trigger enhanced regeneration (Figure 1f), which suggests that CED-3, though necessary, may not be sufficient to promote efficient regeneration. However, because it may be difficult to achieve CED-3 cellular expression levels that permit optimal repair rather than cell death (Figure S2), whether CED-3 might have the capacity to drive regeneration on its own remains unclear.

### CED-3 Acts at Early Steps of Axonal Regeneration

**Figure 2:**
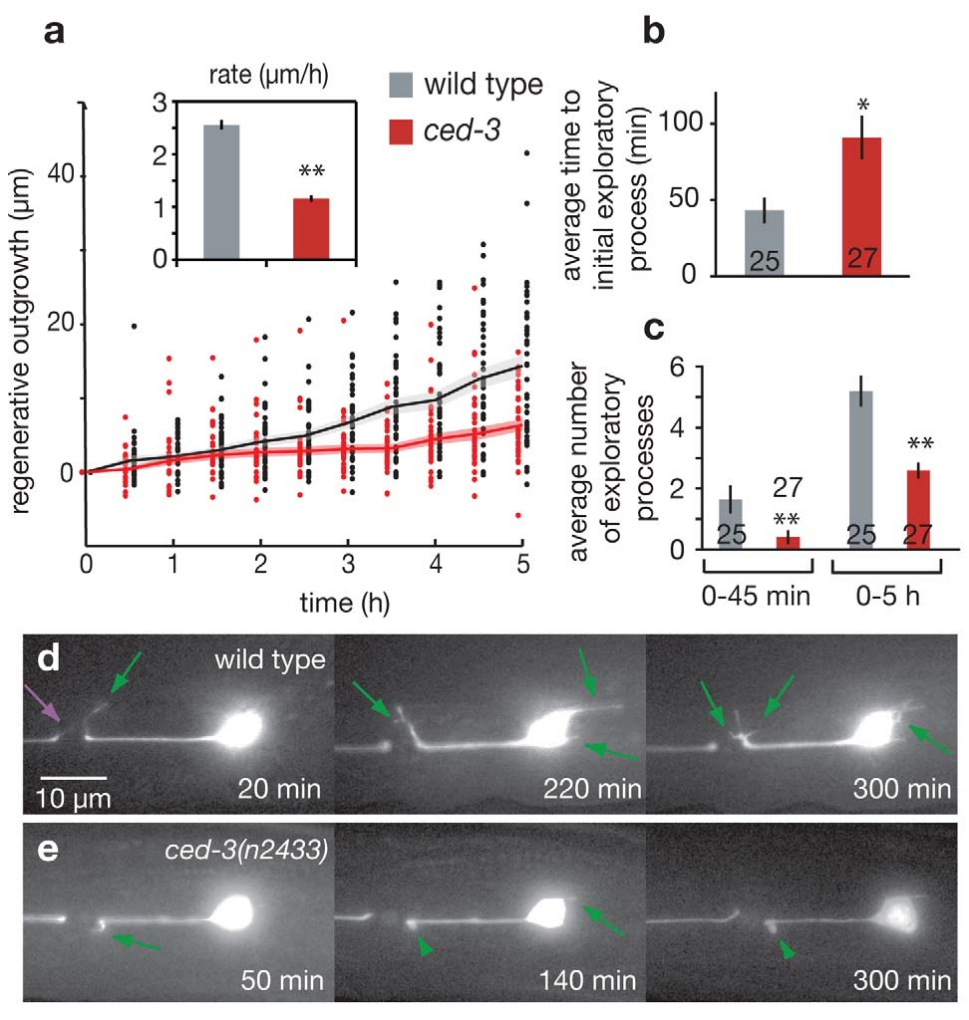
CED-3 caspase contributes to early dynamics of axonal regeneration. (a) Time-lapse regenerative outgrowth measurements during the 0–5 h time period following laser surgery for WT (grey) and *ced-3(n2433)* (red). Data points indicate outgrowth of individual neurons, and lines indicate average outgrowth (shaded region areas ± s.e.m.). The insert shows total outgrowth rates over the 0–5 h time period (calculated using a regression fit of the displayed outgrowth data, restricted to pass through the origin). (b) Mean time of initial outgrowth after laser surgery for WT (grey) and *ced-3(n2433)* (red) mutant worms as determined from time lapse measurements. (c) Mean number of individual exploratory processes generated during the 0–45 min and 0–5 h time periods following laser surgery. (d) Representative images showing numerous exploratory outgrowths, sprouting of small often short-lived processes, in the WT background, compared to (e) relatively few such protrusions in the *ced-3(n2433)* mutant background. Green arrows mark new exploratory processes, green arrowheads mark stunted or stalled processes, purple arrow marks an exploratory process from the disconnected distal axon segment, and time is indicated in minutes post-laser surgery. For bar graphs, data are expressed as mean ± s.e.m. *p<0.05, **p<0.005 versus wild type by Student's *t* test.

To evaluate *ced-3* impact on regeneration in greater detail, we acquired time-lapse images of regrowing neurons for the first 5 h following laser axotomy. We accomplished this using nematode immobilization techniques that are stable over long time periods without the use of harsh anesthetics (see Materials and Methods and Figure S3) [16],[17]. We found that both the rate and extent of new outgrowth were dramatically reduced in *ced-3* mutants during the initial 5 h following laser axotomy, with total outgrowth reduced by ∼45% and the average outgrowth rate reduced by 55% (Figure 2a). Higher resolution analysis of initial regenerative dynamics in WT and *ced-3* mutants revealed three striking phenotypes in regenerating *ced-3* neurons that impact the sprouting of short, often transient, exploratory filipodia-like processes that dominate during this early stage of outgrowth: (1) there is a significant delay in outgrowth onset after axotomy, with first signs of re-growth appearing after 91±13 min on average in *ced-3* mutant axons compared to 43±8 min characteristic of WT axons (Figure 2b); (2) the number of sprouts initiated in *ced-3* mutants is greatly diminished 0–5 h post-surgery, with the greatest effect observed during the initial 0–45 min (Figure 2c); and (3) *ced-3* extensions often appear defective or stunted, resulting in short, wide, persistent bleb-like outgrowths that are distinctly different from the transient, dynamically active filopodia-like extensions of WT neurons (Figure 2d,e, Movies S2 and S3). These dramatic defects in the initiation of regrowth responses to axotomy in *ced-3* contrast with overall outgrowth scores 3 d post-surgery, which no longer show differences from WT (Figure 1d). We conclude that the CED-3 apoptosis caspase impacts very early events in post-axotomy filipodia extension but is not essential to regrowth per se, suggesting that, like in other *C. elegans* regeneration studies [18], additional gene activities may act in parallel to promote regeneration.

*ced-3* mutant neurons are not generally defective in developmental growth cone formation or guidance. In *ced-3* mutants, we observed that developmental growth cones of migrating VD motor neurons in L1 larvae exhibit wild-type behaviors when they contact a new surrounding tissue: rounded in the hypodermis, and anvil-shaped when contacting the lateral nerve cord or body wall muscle cells (Figure S4) [19]. In addition, when we examined the AVM touch neuron projection to the ventral nerve cord (VNC) (a model for in vivo regenerative axon guidance [2]) by laser dissecting the AVM process half way to the VNC, we found that the *ced-3(n2433)* mutant shows the same ability to reach the ventral nerve cord 24 h post-surgery as the wild type (WT: 63%±9.3% reach the VNC, *N* = 27 [2]; *ced-3(n2433)*: 64.3%±9.1%, *N* = 28, no statistical difference by *t* test). Together, these observations suggest that *ced-3* defects in early filopodia extension dynamics and outgrowth might be limited to injury responses, although detailed quantification of developmental outgrowth and guidance needs to be accomplished before relative roles in development versus injury can be definitively assigned.

Interestingly, our high-resolution time lapse studies also revealed that the distal part of the axotomized axon, disconnected from the cell body, exhibited regrowth attempts by blebbing and extending exploratory processes initially similar in appearance to those in the proximal end (Figure 2d purple arrow, Movies S2 and S3). However, in the *ced-3* mutant 0–5 h post-surgery, growth from the distal side of the laser cut was both delayed in onset (58±13 min in WT versus 111±22 min in *ced-3* distal termini, *p*<0.05) and diminished in extent (1.8±0.2 exploratory processes in WT versus 1.3±0.2 in *ced-3*, *p*<0.05) (Figure S5). These initial regenerative responses of axon segments separated from the cell body must therefore be driven by *ced-3* proteins or transcripts already present in the injured axon [20]. Thus, it appears that a nucleus-independent mechanism of CED-3 caspase activation lies in wait in healthy processes prior to injury.

### CED-3 Is Needed for Rapid Reconnection Following Axotomy

**Figure 3:**
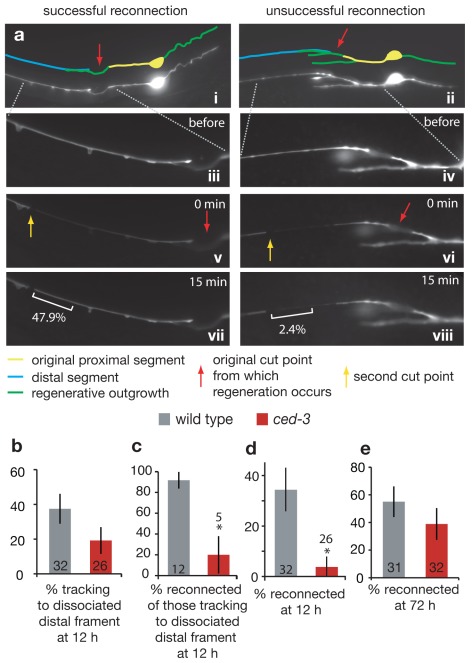
CED-3 caspase contributes to reconnection to the dissociated distal fragment. (a) Photo-bleaching test for successful reconnection (see Materials and Methods for details). (i, ii) Compressed z-stack images 12 h post-laser surgery of neurons displaying apparent reconnection. Red arrow indicates the original cut point. (iii, iv) Magnified image of distal segment (single z-frame). (v, vi) A second laser cut (yellow arrow) is followed by selective photo-bleaching between the two cut points. (vii) Recovery of GFP fluorescence in the original distal segment within 15 min indicates the existence of a fusion between the regenerating proximal axon segment and the distal segment, and (viii) a lack of fluorescence recovery indicates no such reconnection (with a cutoff of <7.7% fluorescence score, see Materials and Methods). White brackets indicate the portion of process analyzed for fluorescence recovery; numbers indicate percent recovery of fluorescence. (b) Percent of re-growing axons that track to the place of the dissociated distal fragment at 12 h (i.e., appear to be in physical contact). Note that although *ced-3* mutant axons tend to track less often to the dissociated distal fragment, the differences are not statistically significant (*p* = 0.217). (c) Percent of neurons, of those that track to the dissociated distal fragment, that are also scored to have reconnected at 12 h. Specific reconnection events (in addition to poor tracking) appear delayed in *ced-3* mutant axons. (d) Percentage of total neurons at 12 h post-surgery, for which the regenerating proximal axon successfully reconnected with the disconnected distal axon segment. (e) Percentage of total neurons at 72 h post-surgery, for which the regenerating proximal axon successfully reconnected with the disconnected distal axon segment. Note that reconnected axons do not show filopodial extensions, suggesting this trait might be suppressed in reconnected neurons as well as in intact neurons. All comparisons are by Fisher's exact test, with *p<0.05.

In *C. elegans*, injured neurons can reconnect to reestablish the cytoplasmic connection of the proximal axon with the dissociated distal region of the axon [8],[13]. To generate a more complete picture of the consequences of *ced-3* deficiency, we assayed regenerative capacities of those WT and *ced-3* mutant neurons that tracked back to the dissociated process (i.e., those not counted for overall outgrowth due to coincidence of old and new processes) using a cytoplasmic reconnection assay. To score for reconnection, we adapted a fluorescence transfer protocol for use with GFP (see Materials and Methods for details) [21]. In our assay, we isolated a segment of the previously severed fragment by introducing a second cut more distal to the initial injury/potential reconnection site; we then selectively photo-bleached GFP within this distal segment (Figure 3a). Rapid recovery of GFP fluorescence within this segment revealed free diffusion of GFP from the non-photobleached regenerating proximal axon into the formerly severed fragment, and thus a re-established cytoplasmic connection.

To assay regeneration phenotypes of those neurons that regrew to come in proximity to the dissociated process, we compared WT and *ced-3* mutant neurons for restored cytoplasmic continuity. We found that *ced-3* mutant neurons were somewhat diminished in their capacity to rapidly track back to the dissociated fragment (Figure 3b), but, of the neurons that grew back to, and appear to be in contact with, the dissociated distal process at 12 h, 92%±8% of WT versus 20%±18% of *ced-3* processes successfully reconnect (*p*<0.05 Fisher's exact test) (Figure 3c). When we sum data for *all* axotomies at 12 h post-surgery, 34%±8% of total WT ALM axons severed were reconnected at this time point, as compared to 4%±4% of *ced-3* mutant axons (Figure 3d). As is true for the outgrowth phenotype, reconnection can approach WT levels after a significant time lag (Figure 3e). We conclude that a consequence of *ced-3* caspase inactivation is delayed reconnection. Although the reconnection defect might be an indirect consequence of slow initial outgrowth, it is clear that CED-3 caspase deficiency impairs both initiation of axonal regeneration and reparative timing. In cultured *Aplysia* neurons, the time to reconnection can influence long-term function of the neuron [22], so the speed to reconnection might hold physiological relevance in invertebrate physiology.

### CED-4/Apaf-1, But No Other *C. elegans* Apoptosis Regulator, Is Required for Efficient Regeneration

**Figure 4:**
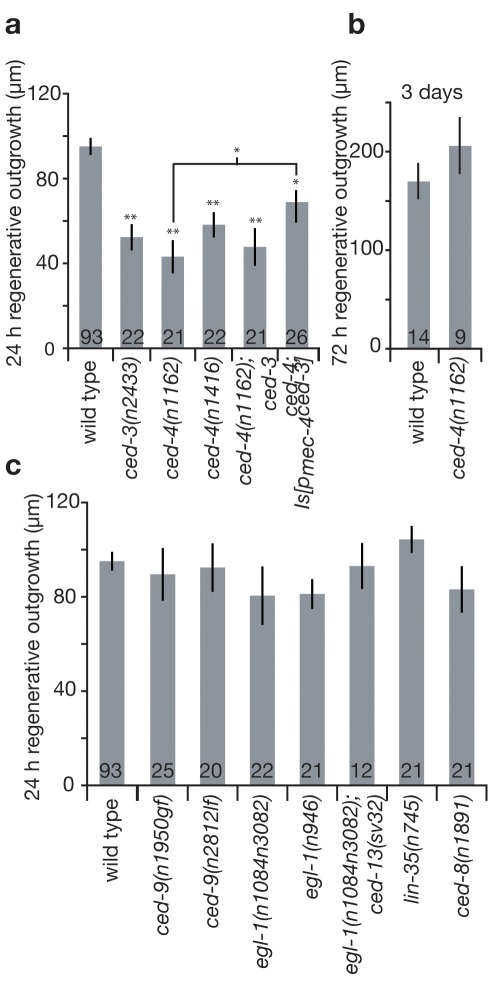
The *ced-4* core apoptotic gene, but not known *C. elegans* upstream regulators of developmental, germline, or radiation-induced apoptosis, are needed for efficient axonal regeneration. (a) We measured mean regenerative outgrowth in ALM neurons 24 h after laser surgery for WT and mutant strains affecting *ced-3* and *ced-4* (two independent alleles) core apoptotic genes, compound mutants, and in *ced-4; Is[p_mec-4_ced-3]*. Because some studies [9],[10],[18] documented mutant neurons that show virtually no post-axotomy regeneration and we find that the *kgb-1 ced-3* double mutant exhibits lower regeneration than the *ced-3* single mutant (Figure S6), the partial phenotype can become more severe in compound mutants. (b) Regenerative outgrowth was measured 3 d after surgery in ALM neurons in WT and *ced-4(n1162)* (no statistical difference by *t* test). (c) Regenerative outgrowth was measured 24 h after surgery in ALM neurons for mutants in upstream apoptosis regulators as well as compound mutants (which show no statistical difference by one-way ANOVA test). Bar graphs depict mean ± s.e.m. For (a), the Student's *t* test, with a Dunn-Sidak adjustment for multiple comparisons, was used to determine the statistical significance of differences versus WT, with brackets indicating direct Student's *t* test between two specific values. *p<0.05, **p<0.005.

A pressing question raised by the discovery of the role of CED-3 caspase in post-axotomy neuronal responses is whether other apoptotic pathway components modulate neuronal regeneration. During *C. elegans* developmental apoptosis, the expression of EGL-1 (BH3 domain only protein) inhibits CED-9 (Bcl-2 family member), releasing CED-4 (apoptosis protease activating factor-1 Apaf-1 homolog), which in turn activates CED-3 caspase [23]; CED-8 modulates the timing of developmental apoptosis [24]. Physiological germline apoptosis requires *ced-9* transcription directed by the *lin-35* Rb ortholog [25], and under conditions of radiation stress, both the *C. elegans* BH3-only domain proteins EGL-1 and CED-13 are needed for CED-3-dependent apoptosis [26]. To address how CED-3 caspase might be activated by axotomy, we tested roles of known apoptosis regulators in regeneration using the amount of 24 h outgrowth as a measure. We found that *ced-4(n1162)* and *ced-4(n1416)* mutants displayed diminished regeneration similar to *ced-3(n2433)*, establishing that CED-4 functions in axonal regeneration as well as in apoptosis (Figure 4a). The double mutant *ced-4(n1162); ced-3(n2433)* is impacted to the same degree as either single mutant, suggesting that *ced-3* and *ced-4* work in the same pathway to influence regenerative outgrowth (Figure 4a). We also found that expression of our one minimally toxic *ced-3* transgene in the touch neurons partially rescued the *ced-4(n1162)* defect, consistent with *ced-3* acting downstream of *ced-4* in axonal regeneration (the same as the order of CED-4 and CED-3 action in apoptosis) (Figure 4a). As with *ced-3*, regenerative defects of *ced-4* mutant animals were no longer apparent after 3 d (Figure 4b). We conclude that *ced-4* is needed for efficient regeneration and acts upstream in the same pathway as *ced-3*.

Other known upstream regulators of apoptosis, including loss-of-function *(lf)* allele *ced-9*(*n2812*), gain-of-function *(gf)* allele *ced-9(n1950)*, *egl-1 lf* mutants *egl-1(n1084n3082)* and *egl-1(n986)*, the *egl-1; ced-13* double mutant lacking both *C. elegans* BH3-only domain proteins, and *lin-35(n745)*, did not affect regeneration proficiency, revealing an alternative regulatory mechanism for CED-4 and CED-3 activation in the response to axotomy (Figure 4c). Likewise, because the *ced-8(n1891)* mutation did not impact regeneration, we conclude that the delayed regeneration response in *ced-3* mutants is unlikely to be the consequence of timing-regulator *ced-8* action in axonal regeneration. Overall, our data reveal an unexpected reconstructive role for the core apoptotic proteins CED-3 and CED-4 that is mobilized via a novel regulatory mechanism distinct from known apoptosis regulatory pathways.

### DLK-1 Kinase and CED-3 Appear to Act in the Same Pathway to Promote Regeneration

**Figure 5:**
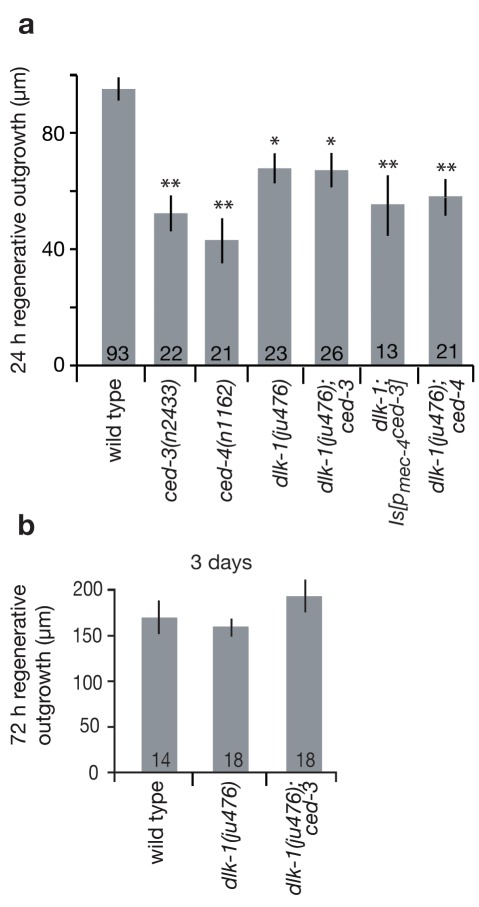
The MAPKKK *dlk-1* might act together with *ced-3* to promote regeneration. (a) Mean regenerative outgrowth in ALM neurons was measured 24 h after laser surgery for WT and mutant strains for *ced-3*, *ced-4*, *dlk-1*, double mutants *dlk-1; ced-3* and *dlk-1; ced-4*, as well as *dlk-1; Is[p_mec-4_ced-3* *]*. Note that double mutant *kgb-1 ced-3* exhibits lower regeneration than *ced-3* alone (Figure S6), as would be predicted for action in a parallel regeneration pathway, so it is experimentally possible for a double mutant to exhibit lower regeneration. (b) Regenerative outgrowth in ALM neurons was measured 3 d after laser surgery for WT and mutant strains *dlk-1* and *dlk-1; ced-3*. Bar graphs depict mean ± s.e.m. For (a), the Student's *t* test, with a Dunn-Sidak adjustment for multiple comparison, was used to determine the statistical significance of differences versus WT in each panel; *p<0.05, **p<0.005. For (b) there is no statistical difference by one-way ANOVA.

The DLK-1 p38-like MAPK pathway has been shown to play a critical role in *C. elegans* neuronal regeneration [8]–[10]. Our detailed phenotypic analysis of *ced-3* suggests action early in axonal regeneration, influencing initial exploratory sprouting (Figure 2), and similarly, the *dlk-1* mutant has a drastic reduction in primary growth cone formation consequent to axotomy [9]. We therefore addressed whether DLK-1 might act together with CED-3 and CED-4 in the same molecular pathway, or alternatively, might act in parallel. Using our femtosecond laser and immobilization protocol, we find that the single mutant *dlk-1(ju476)* displays ALM regenerative outgrowth similar to that of *ced-3* mutants, with a ∼50% reduction as measured at the 24-h time-point but wild-type regeneration proficiency at 3 d (Figure 5a,b). In the *dlk-1(ju476)* mutant background, weak regeneration of touch neurons severed in the adult contrasts with total block of regeneration of D-type motoneurons severed at the L4 larval stage, as we measured no regeneration outgrowth following axotomy in 22/22 D-type motoneurons (unpublished data) [8]–[10], underscoring that different molecular mechanisms might control regeneration in different cell types or developmental stages and, more specifically, that multiple redundant pathways may influence regeneration in adult ALM neurons. Interestingly, the double mutant *dlk-1(ju476); ced-3(n2433)* exhibited ALM regeneration impairment similar to that of single mutants, both at 24 h and at 3 d post-surgery (Figure 5a,b), suggesting action in the same pathway. Additionally, the double mutant *dlk-1(ju476); ced-4(n1162)* showed the same regeneration defect as the single mutants at 24 h (Figure 5a), further genetic evidence in support of action in the same pathway. Finally, expression of *ced-3* in the touch neurons did not ameliorate regeneration deficiencies in *dlk-1* mutants (Figure 5a), suggesting that *ced-3* may act upstream of *dlk-1* to promote early events in regeneration of ALM touch neurons in adult *C. elegans*.

Kinase KGB-1 of the (JNK) MAPK pathway has recently been shown to operate in parallel to DLK-1 to promote axon regeneration [18]. We find that although mutant *kbg-1(um3)* is defective in ALM regeneration, the double mutant *kgb-1(um3) ced-3(n2433)* is significantly more impaired in overall regrowth scores than either of the *kgb-1* or *ced-3* single mutants (Figure S6). Our data suggest that, similar to *dlk-1*, *ced-3* acts in a separate regeneration pathway from *kgb-1*. Together, these studies define two parallel processes, one involving *ced-4*, *ced-3*, and *dlk-1*, and the other involving *kgb-1*, that act in ALM axon regeneration.

### CED-3 Caspase Promotes Axonal Regeneration in a Calreticulin-, Calcium-Dependent Pathway

**Figure 6:**
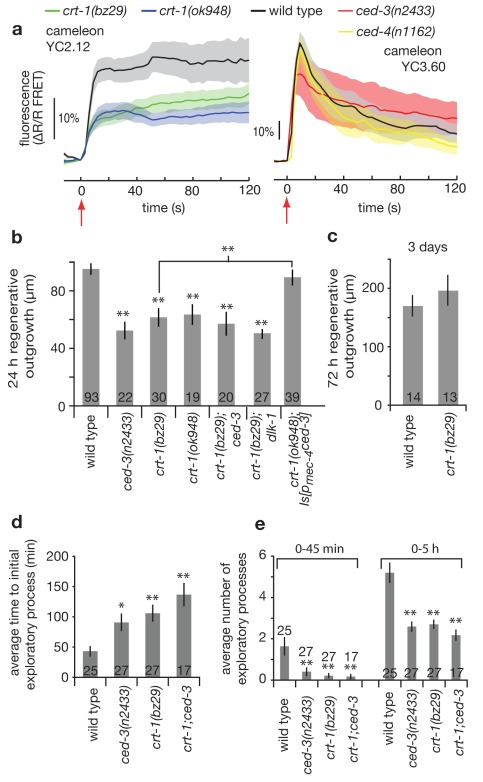
CED-3 caspase acts in a calreticulin-, calcium-dependent pathway for efficient axonal regeneration. (a) Intracellular calcium dynamics in the ALM neurons during laser axotomy. Two different variants of the FRET-based calcium-sensitive fluorophore cameleon were used: Left panel, YC2.12; right panel, YC3.60. Differences in the wild type response (amplitude and shape) are due in part to the lower calcium affinity and larger dynamic range of the YC3.60 fluorophore compared to that of YC2.12 [58]. All laser axotomies were performed 20 µm from the cell soma at time = 0 s (red arrow). Traces represent average response at the cell soma (≥9 trials per trace), and shaded regions indicate s.e.m. (b) Mean regenerative outgrowth in ALM neurons measured 24 h after laser surgery for the indicated mutant strains and compound mutant strains defective in the ER calcium-binding chaperone calreticulin, including in the context of *ced-3* expression in touch neurons (indicated as *crt-1; Is[p_mec-4_ced-3]*). (c) Regenerative outgrowth was measured 3 d after surgery in ALM neurons in *crt-1(bz29)* (no statistical difference was found by Student's *t* test). *crt-1(bz29)* and *ced-3(n2433); crt-1(bz29)* double mutant were compared with WT and *ced-3*. (d) Mean time of initial outgrowth after laser surgery, and (e) mean number of individual exploratory processes generated during the 0–45 min and 0–5 h time periods following laser surgery. Bar graphs depict mean ± s.e.m. For (b), (c), (d), and (e) the Student's *t* test, with a Dunn-Sidak adjustment for multiple comparison, was used to determine the statistical significance of differences versus WT in each panel, with brackets indicating direct Student's *t* test between two specific values; *p<0.05, **p<0.005.

Calcium signaling is known to play a fundamental role in the neuronal responses to damage and subsequent recovery, with acute cellular insult inducing large intracellular calcium transients important for regrowth [7],[8]. To address whether calcium signaling could play a role in the CED-3/CED-4 molecular pathway during regeneration, we performed in vivo measurements of cytoplasmic calcium levels in the touch neuron cell soma during laser axotomy using two versions of the genetically encoded fluorophore cameleon (see Materials and Methods). Laser axotomy of WT neurons initiates an immediate (within <3 s) and dramatic increase of cellular calcium levels reported by cameleon-based FRET (Figure 6a). In two independent *crt-1* mutants, which lack the ER calcium-binding chaperone calreticulin known to contribute to cellular calcium homeostasis, we found neuronal damage-induced calcium signals are reduced by ∼50% (Figure 6a, left panel). By contrast, no dramatic defect in calcium responses was detected in either *ced-3(n2433)* or *ced-4(n1162)* mutants as compared to WT (Figure 6a, right panel). Thus, ER calcium stores modulated by CRT-1 influence early calcium fluxes in response to axotomy, but CED-3 and CED-4 do not influence early calcium changes in the injured neuron.

To genetically address the requirement for calcium in regeneration, we tested the *crt-1* mutants for total regenerative outgrowth and found a significant deficiency for both at the 24 h time point, which was no longer apparent after 3 d (Figure 6b,c). We noted that cytoplasmic expression of calcium-binding cameleon YC2.12, which might sequester some intracellular calcium, diminished overall outgrowth, consistent with a role for calcium in directing re-growth responses (Figure S7). By contrast, expression of cameleon YC3.60 that has a lower calcium binding affinity does not appear to affect regeneration (see Materials and Methods). Note that despite a dampening effect of cameleon YC2.12 on total regenerative outgrowth, the relative differences between WT and *crt-1* mutants in regeneration of ALM mechanosensory neurons 24 h post-surgery were maintained in cameleon-expressing lines.

We examined early regeneration phenotypes in *crt-1(bz29)* using high resolution video analysis and found that, similar to *ced-3*, the first signs of re-growth in axotomized *crt-1* mutant neurons appeared with a significant delay (Figure 6d) and that numbers of exploratory processes were highly reduced over both the 0–45 min and 0–5 h time periods post-axotomy (Figure 6e). Our combined genetic and imaging results implicate calcium changes that are activated by injury, and dependent upon calreticulin, in initiation of regeneration. Data follow recent findings that correlate reduced calcium transients resulting from nerve damage with diminished neuronal regeneration in *C. elegans* (see note in Materials and Methods on some experimental differences) [8].

To probe the relationship between *crt-1* and *ced-3* in regeneration, we compared regenerative capacity in the *ced-3(n2433)* single mutant, the *crt-1(bz29)* single mutant, and the *ced-3(n2433)*; *crt-1(bz29)* double mutant. We find that regeneration deficits at 5 h and at 24 h in the double mutant were similar to those in single mutants, consistent with the possibility that CED-3 and CRT-1 act via the same pathway to influence initiation of regeneration (Figure 6b,d,e). We also found that expression of our one minimally toxic *ced-3* transgene in the touch neurons partially rescued the *crt-1(bz29)* defect (Figure 6b), suggesting that CRT-1/calcium elevation might act upstream of CED-3 activation during axonal regeneration. This is in agreement with the calcium imaging data in Figure 6a showing a defect in calcium signaling in the *crt-1* mutants but not in the *ced-3* or *ced-4* mutants. Finally, the double mutant *dlk-1; crt-1* showed similar defects to the single mutants (Figure 6b), consistent with the action of *dlk-1* in the same pathway as *crt-1/ced-4/ced-3*. Taken together, our data are consistent with a model in which *crt-1* could act to influence intracellular calcium signals needed for CED-4-dependent localized CED-3 activation and efficient regeneration initiation promoted in part via kinase DLK-1.

## Discussion

Here we document novel roles of core apoptosis executors in the initiation of process regrowth in axotomized neurons. CED-3 caspase activity within the injured neuron promotes rapid remodeling and outgrowth, often resulting in efficient reconnection. *C. elegans* apoptosis executor CED-3 contributes to early regenerative events via a process genetically implicated to include CED-4 and calreticulin. The DLK-1 kinase might act downstream in the *crt-1/ced-4/ced-3* pathway. The definition of reconstructive roles for the core apoptosis executor CED-3 holds implications for regenerative medicine strategies.

### Caspase CED-3 Is Needed for Early Regrowth and Appears Ready for Rapid Activation

High resolution video microscopy time course studies during the first 5 h post-axotomy revealed that in wild type proximal processes, distinctive filopodia-like extensions can appear within minutes, leading to active growth cones and extensive outgrowth. In *ced-3*, these responses are slowed and often appear markedly defective—*ced-3* processes take longer to initiate outgrowth, there are fewer filopodia generated, and there is less overall outgrowth. One particularly striking phenotype is that severed processes in *ced-3* mutants can appear to produce extensions that do not mature into filopodia—instead, ends persist as rounded blebs that lack structure and do not extend (Movie S3, Figure 2e). Previous in vitro screens have identified *C. elegans* cytoskeletal proteins, such as actin, tubulin, and myosin chains, as potential CED-3 targets [27]; and caspases can cleave mammalian cytoskeletal proteins and their regulators [28],[29]. Thus, although critical targets in the regrowth mechanism remain to be identified, one possibility is that CED-3 activity might induce structural rearrangements needed for efficient filopodia production by cleaving cytoskeletal proteins.

Eventually, both total regenerative outgrowth and reconnection to the severed distal fragment reached WT levels at longer time points in *ced-3* mutants, 3 d post-surgery (Figure 1d and Figure 3e). This outcome is consistent with a model in which the CED-3 caspase plays a role in the kinetics of a single regeneration pathway; alternatively, other pathways may run in parallel to promote regeneration and these other pathways may eventually compensate for *ced-3* defects. Given the complex processes that influence regeneration in *C. elegans* [6],[18] and mammalian systems [30], and our genetic data that suggest kinase KGB-1 acts in parallel to caspase CED-3, the contribution of multiple pathways to regeneration seems like a probable scenario. The dramatic deficits in the initiation and early outgrowth dynamics suggest that CED-3 plays a prominent role during this critical stage of regeneration. Because *dlk-1* is needed for early growth cone formation [9], it exhibits similar outgrowth defects to *ced-3*, the *dlk-1 ced-3* double mutant shows similar regenerative outgrowth defects as single mutants, and elevated expression of *ced-3* does not ameliorate the *dlk-1* mutant deficit, we suggest that conserved kinase DLK-1 may be an integral downstream component of this early-acting mechanism.

### A Caspase Ready for Repair

Interestingly, our studies reveal that both the proximal process (remaining in contact with the nucleus) and the dissociated distal process (devoid of a nucleus) exhibit early regrowth efforts, generating dynamic filopodial extensions. Changes in the dissociated end have been noted in another *C. elegans* regeneration study [10]. As growth cones have been observed to extend from isolated processes in injured cultured vertebrate neurons [31]–[33], this phenomenon might represent another conserved element of the injury response. We find that in *C. elegans*, the regenerative response in the dissociated end is significantly diminished when *ced-3* is lacking, and thus *ced-3*-dependent responses can occur independently of a nucleus and new transcription. These observations suggest that CED-3 protein might persist at low levels in an inactive form in healthy axons, evidence for which has been previously noted in touch neurons [15] and suggested for other non-apoptotic caspase paradigms [34]. Low basal level caspase activity can modulate motility in some cell types [35],[36] and might contribute to regeneration in this case. Alternatively, *ced-3* transcript distributed throughout healthy processes might be translated at the injury site upon transection, as rapid local translation of other messages has been documented at injury sites in in vitro mammalian culture models [20] and in *C. elegans* [10] (including *dlk-1*). Regardless of activation strategy, it appears that *C. elegans* neurons can rapidly employ CED-3 activity when regenerative repair growth is needed.

### CED-3 Is Needed for Rapid Reconnection

Wild type regenerating *C. elegans* neurons are capable of rapidly locating and re-fusing with the dissociated distal process (34.4% successfully reconnected at 12 h; >50% reconnected by 72 h). We observed that *ced-3* mutant processes are diminished in reconnection at the 12-h time point—fewer neurons overall reconnect (3.8% for *ced-3* versus 34.4% for WT), and of neurons that do successfully track to the distal severed process, fewer *ced-3* ends successfully reconnect (20.0% for *ced-3* versus 91.7% for WT). Eventually, severed neurons do grow and reconnect in the *ced-3* mutant background. Thus, *ced-3* is not essential for reconnection, but rather plays a role in promoting rapid reconnection. The phenomenon of reconnection raises the question as to whether process breaking is a natural in vivo challenge in the development and/or function of neurons such that a protective mechanism of repair has evolved. Interestingly, severed *Aplysia* neurons also reconnect in culture, with failure to reconnect associated with electrophysiological dysfunction of the proximal neuron [22]. Rapid reconnection might thus be physiologically important for restoring or maintaining the function of the injured neuron.

### Defined Pathways That Regulate Apoptosis Are Not Operative in Regeneration

We tested multiple regulators of *C. elegans* somatic or germline apoptosis for an effect on neuronal regeneration but find that regrowth is not influenced by CED-9/Bcl-2, BH3 domain proteins EGL-1 and CED-13, or the LIN-35 germline apoptosis regulator. Likewise, regeneration responses are not altered in a *ced-8* mutant in which the progression through apoptosis is slowed. These data support that CED-3 caspase must be regulated by a novel mechanism that transpires independently of known apoptosis regulatory pathways.

The one other apoptosis protein needed for efficient regeneration is Apaf-1/CED-4, which our genetic analysis suggests acts upstream in the same pathway as *ced-3*. In apoptosis, CED-4 oligomerizes to form the apoptosome structure that facilitates procaspase cleavage [37]. It is possible that a similar reaction occurs in the regenerative response, although this process would likely need to be tightly regulated to prevent apoptosis (see below). *ced-4* has been documented to execute some functions independently of *ced-3* [38]–[40], and one instance of non-apoptotic cell death in neurons knocked down for mitochondrial coenzyme Q involves both CED-3 and CED-4 [41]. To our knowledge, however, our finding is the first report of CED-3 and CED-4 co-function in a pro-survival mechanism.

### A Working Model for Calcium-Dependent CED-4 Activation of CED-3 for Regenerative Outgrowth

If CED-4 activates CED-3 caspase activity, a key question becomes how CED-4 might become proficient to do so consequent to axotomy. Interestingly, the CED-4 protein contains two regions that exhibit similarities to EF-hand calcium binding domains [42]. Our data and that of others [8] document local and transient elevation of calcium within the damaged neuron, and also show that limiting calcium signals from the ER or plasma membrane can diminish regeneration. Thus, one model for CED-3 activation in regeneration could be that calcium transients resulting from nerve damage, amplified by CRT-1, might locally activate CED-4, which in turn activates CED-3. Consistent with this model, we find that calcium dynamics in response to axotomy are disrupted in *crt-1* mutants, but are normal in *ced-4* and *ced-3* mutants.

### Limiting Caspase Activity to Localized Repair Rather Than Cell Destruction

Once CED-3 becomes activated, its proteolytic functions must be tightly regulated to prevent apoptosis. Indeed, the need for a delicate balance is evident by the extreme difficulty and resulting cell death that we and others have encountered with introducing caspase transgenes, which most often kills cells (Figure S2) [15]. If maintained high calcium is needed for continued activation, local calcium transients initiated by membrane lesion might confer regulation; it is also possible that a mechanism exists for very low level basal level activity [35],[36]. Two elegant examples of localized caspase activation/regulation for developmental functions in *Drosophila* are the pruning of dendrites in the restructuring nervous system [43],[44] and the differentiation of spermatids [45]. Mammalian caspases have been shown to function in cell differentiation, cell migration, olfactory neuron development [46], and modulation of long-term depression in the brain [47]. Our findings on CED-3 and CED-4 roles in axon repair extend thinking on how proteins known to orchestrate apoptotic cell death can also contribute to pro-life functions [45].

### CED-3 Repairs Axons: Implications for the Treatment of Nerve Injury

CED-3 is the executor caspase for all *C. elegans* apoptosis, yet CED-3 clearly influences regenerative neuronal repair. The growth protein GAP-43 and the transcription factors p53 and c-jun can have dual roles in both promoting neuronal death and regeneration following axonal injury [48], raising the possibility that recruitment of cell death machinery in localized axonal regrowth might be a feature shared across phyla. Indeed, our data intersect with independent findings in culture models that suggest a mechanism similar to what we propose for *C. elegans* regeneration might influence regrowth in vertebrate neurons. Studies on in vitro vertebrate neuronal culture showed that caspase-3 is rapidly activated within 5 min of application of the guidance cues netrin-1 and LPA on growth cones to promote chemotropic responses [49] and that addition of caspase-3 inhibitors hinder growth cone formation after axotomy [20]. Moreover, calreticulin expression has been found to be dramatically induced in mammalian growth cones [50]. Together, these studies raise the possibility that localized deployment of caspases and calreticulin activity in axonal regeneration may be conserved in higher organisms. If caspases are found to promote mammalian axonal regeneration, regulated activation of caspase-promoted regrowth/reconnection might be used to promote functional repair in regenerative microsurgery or in injury therapy; at the same time, the use of anti-caspase therapeutics to limit neuronal loss following nerve damage [51] might be reconsidered.

## Materials and Methods

### Laser Microsurgery and Microscopy Techniques

Laser surgery was performed as described earlier [52]. A Ti:sapphire laser system (Cascade Laser, Eclipse Pulse Picker, KMLabs, Boulder, CO or Mantis PulseSwitch Laser systems Coherent Inc., Santa Clara, CA) generated a 1 kHz train of ∼100 fs pulses in the near infrared (∼800 nm). The beam, focused to a diffraction limited spot (using either a Nikon 100×, or 60×, 1.4 N.A. microscope objective), resulted in vaporization and tissue disruption with pulse energies ranging from 5–15 nJ. Visual inspection of the targeted neuron immediately following brief laser exposure (∼100–500 ms) confirmed successful axotomy. In some cases multiple laser exposures were necessary to generate a visual break in the nerve fiber. For 24 h regeneration measurements, *C. elegans* were temporarily anesthetized on 2% agar pads containing 3 mM sodium azide to allow for laser surgery, subsequently rescued, and then re-anesthetized 24 h later for imaging.

### Regenerative Outgrowth Measurements

We targeted ALM axons 20 µm from the cell body unless otherwise stated and D-type motor neurons 20 µm up from the ventral nerve cord along the ventral-dorsal commissure. For length measurements, we calculated the total outgrowth of a neuron by summing lengths of the multiple outgrowth branches (for example, the green traces in Figure 1av), excluding very short branches (those <5 µm long). For ALM 24 h and 72 h regeneration outgrowth analysis (Figures 1, 4, 5, and 6 and Figures S6 and S7), regrowth of the proximal end only was monitored. The outgrowth of the distal end was measured only in data presented in Figure S4. The integrated lines *zdIs5[p_mec-4_gfp]* [53] and *rnyIs014[p_mec-4_mCherry unc-119(+)]*, a gift from K. Nehrke, U. Rochester Medical School, were used for ALM surgeries in young adults. D-motor neuron surgeries were performed in L4 larvae using *oxIs12[p_unc-47_gfp]* [54]. Note, at 72 h, outgrowth measurements in different genetic backgrounds (data in Figures 1d, 4b, 5b, and 6c) showed no statistical difference from one another by one-way ANOVA.

About 35% of WT axons reconnect within 24 h. For scoring of regenerative growth, we focused on instances in which we could get an accurate measurement of the total length and *excluded* these potential reconnection events from outgrowth scores, as in those cases we could not distinguish new growth from old process (the old process persists and does not lose GFP signal). Wild-type data were generated in six distinct groups, taken months apart, in strains ZB154 *(zdIs5[p_mec-4_gfp])* and KWN177 *(rnyIs014[p_mec-4_mCherry unc-119(+)])* (each group consisting of experiments run on the same or adjacent days using the same reagents). We found no significant difference between 24 h regenerative outgrowth of different WT groups and different transgenic markers (by one-way ANOVA). Wild-type data from all groups were therefore pooled together to give the wild-type measurement reported in the figures.

As measured in the time-lapse analysis (see below), the effect of the *ced-3* mutation in the first 5 h is quite striking, featuring a deficit in exploratory processes, stunted sprout morphology not seen in WT regenerating neurons, and a general delay in response. Although conducting 5 h scoring in all genetic studies might have maximized phenotypic differences, the 24 h time point was used to evaluate relative regeneration in most genetic comparisons (most of these studies were conducted prior to the 5 h measurements that revealed early action of *ced-3*). Because statistically significant differences were still apparent at 24 h, and 5 h high resolution video microscopy required laborious analysis of individual movies, it would have been impractical to redo all genetic analyses for the 0–5 h time points.

Light touch to the anterior part of *C. elegans* body is sensed through a pair of ALM neurons and the AVM neuron. Interestingly, when both ALM axons were cut 20 µm from the cell body (*n* = 10) and AVM was ablated, we found that anterior touch was not significantly reduced, suggesting that the severed distal processes, in contact with post-synaptic interneurons to mediate the escape behavior, maintain the capacity for touch transmission. When we cut both ALM axons >200 µm from the cell body (just posterior to the nerve ring, where critical contacts to interneuron targets are concentrated), touch sensitivity was diminished. Although some axons from this axotomy distance seemed to have a directed regeneration, we did not find evidence of restored touch sensitivity even several days post-axotomy, consistent with previous reports [12].

Some of our scores of extent of regeneration defects differ quantitatively from some published studies. Differences may be attributed to a number of factors, including different anesthetic techniques, neuron type studied, age, and laser surgery technique [2],[8],[9],[12]. A few teams previously reported a delay of ∼10 h in the formation of the growth cone [2],[12]. However, using nematode immobilization techniques that do not require harsh anesthetics (microfluidic devices (Figure S3), as well as a 10% agarose preparation, see below), we observed no such delay: neurons often displayed initial growth within minutes of the laser damage with average initial growth time <1 h (Figure 2b). We observed more robust regeneration in ALM neurons compared to that of D-motor neurons, which may explain some of our differing results with published *dlk-1* mutant strains (we consistently find reduction to ∼50% 24 h regrowth rather than no regrowth; minimal but non-zero regeneration in *dlk-1* mutant strains has been reported in the PLM neurons in other studies [8],[10]). Our laser ablation technique utilizing a 1 kHz femtosecond pulse train at ∼800 nm is specifically designed to deliver precise ablation with minimal collateral damage to the animal and target neuron. Other techniques using MHz femtosecond pulse trains and conventional UV lasers produce larger regions of ablation and therefore more significant damage to the targeted neuron. Although some studies have indicated that postsurgical neuronal regeneration is unaffected by laser ablation technique [12], under certain conditions this may not hold true, leading to possible discrepancies in details among experiments. Despite these technical differences in the field and quantitative differences in extent of regeneration reported, basic conclusions have held across the field.

### Reconnection Test

A significant proportion of axotomized neurons grew back to the dissociated fragment and could not be monitored for total outgrowth. To determine how *ced-3* caspase disruption altered regeneration outcomes in this fraction of axotomized neurons, we scored for reconnection. ALM axons were severed 20 µm from the cell body in young adults using an *zdIs5[p_mec-4_gfp]* marker to visualize processes. 12 h, 24 h, or 72 h post-surgery, neurons were inspected by eye. Neurons for which the regenerative outgrowth of the proximal axon segment appeared to track to (i.e., be in close contact with) the dissociated distal segment (Figure 3a) were further assayed for reconnection using the following photo-bleaching experiment: (a) An initial image of the neuron was recorded (frame 1, see Figure 3a, panels iii and iv). (b) Using the laser, a second cut (yellow arrow in Figure 3a, panels v and vi) was made along the distal segment ∼40 µm from the initial cut point (red arrow) and ∼20 µm from any potential reconnection points. This effectively isolated the distal segment, where there is potential reconnection, from the rest of the process. This was important to prevent GFP refilling from the distal side. (c) The relevant segment (i.e., between the two cut points) was selectively bleached using standard high intensity UV illumination and a restricted illumination field. A second image was acquired immediately after bleaching (frame 2, see Figure 3a, panels v and vi). (d) After 15 min a third picture was acquired (frame 3, see Figure 3a, panels vii and viii).

GFP fluorescence level in each frame was measured as the average intensity along ∼15 µm of the nerve process starting at the second cut point (white brackets in Figure 3a, panels vii and viii), minus the background fluorescence measured adjacent to the nerve process (note, the same portion of the process was analyzed in each successive frame). Percent recovery was calculated as intensity increase between frames 3 and 2, relative to the intensity decrease between frames 1 and 2: percentage recovery = (if3–if2)/(if1–if2) (where if1 is the fluorescence intensity measured in frame 1, etc.). Over the short recovery time, recovery of GFP intensity indicates diffusion of non-bleached GFP into the isolated segment through a new connection point with the regenerating neuron (Figure 3a, panel vii). If there is no reconnection, the segment is truly isolated and GFP fluorescence does not recover (Figure 3a, panel viii). Control experiments, performed by severing the axon of an intact neuron twice and immediately photo-bleaching the isolated unconnected segment, gave an average “recovery” background after 15 min of 3.05%±0.55%. We therefore set a cutoff for successful reconnection at >7.37% recovery (2 sigma from the control average). The percent of reconnection at 12 h as well as additional measurements are given in Figure 3b–d.

### Time-Lapse Imaging

Time-lapse movies following laser surgery were acquired using two methods of worm immobilization: (1) microfluidic devices, the design, fabrication, and use of which followed previously described methods [17], and (2) a preparation of stiff 10% agarose pads and polystyrene microspheres as described earlier [16]. Laser surgery was performed by manual alignment, but subsequent imaging was computer-automated to allow simultaneous time-lapse imaging of up to 10 regenerating neurons in separate *C. elegans*. Initially movies were generated using microfluidic devices at lower resolution (30 min/frame, ×40 magnification). These data were eventually pooled with that from higher resolution movies (see below) to generate the time-lapse outgrowth data shown in Figure 2a (*n* = 43 for WT, *n* = 40 for *ced-3*). For all movies, outgrowth in each frame was measured as the contour length along the new axon growth, with branches <1 µm long excluded. At each time point, mean outgrowth values were calculated across all regenerating neurons of that strain type. Regression fits to the data displayed in Figure 2a (by the least squared error method, KaleidaGraph, Synergy Software, and restricted to pass through the origin) were used to generate the outgrowth rates displayed in the insert. These rates therefore measure the average total outgrowth of the neurons, which at this stage is largely dominated by the creation and retraction of numerous filopodial extensions rather than the elongation of an individual branch.

To generate an accurate account of the initial regenerative dynamics (the number and timing of exploratory processes displayed in Figure 2b–e, Figure 6d,e and Movies S2–S3), higher resolution movies (10 min/frame or 15 min/frame, ×60 magnification) were generated in two ways. Microfluidic devices were used as described above, with the addition of 0.05% tetramisole in the surrounding buffer. The tetramisole worked to partially paralyze the worms [19] in order to keep them still enough for automated re-imaging under high magnification for long time periods. Worms were also immobilized for imaging without anesthetics, using stiff 10% agarose pads and polystyrene microspheres [16]. Data were collected for 5 h post-surgery and images were analyzed by eye (counting number and timing of exploratory processes). Figure 2b,c and Figure 6d,e show the results of data pooled together from the two preparations, as no statistical difference was found between results from the microfluidic devices and stiff agarose protocols.

### Fluorescence Calcium Imaging

We quantified calcium dynamics as changes in ratiometric fluorescence emission between the cyan and yellow fluorescent protein components of cameleon, in the same manner as described previously [55],[56]. Two versions of cameleon were employed: YC2.12 [57] and YC3.60 [56],[58]. For measurements within the *crt-1* mutants we used the *bzIs17[p_mec-4_YC2.12+lin-15(+)]* allele expressing cameleon YC2.12 from the *mec-4* promoter [57]. Because of apparent close linkage between the *ced-3* and the *bzIs17[p_mec-4_YC2.12+lin-15(+)]* allele, we used a second allele expressing cameleon YC3.60 under the *mec-4* promoter, *bzIs158[p_mec-4_YC3.60]*, for measurements in the *ced-3* and in the *ced-4* mutant backgrounds. Images were taken every 3 s with a 300 ms exposure time. The response of an individual neuron was measured as an integration of the fluorescence signal across the entire cell soma. For the YC2.12 measurements, animals were immobilized on a 2% agar pad containing 0.05% tetramisole. For the YC3.60 measurements, the 10% agarose preparation, described above, was used. Differences in the wild type calcium response between YC2.12- and YC3.60-expressing strains could be due to a number of factors including the larger dynamic range and lower calcium affinity of YC3.60, and the different worm immobilization techniques. For these reasons we compared calcium measurements only across genetic backgrounds expressing the same cameleon variant. Likewise, our measured intracellular calcium signals differ with that of others [8] due to a number of possibilities including differing neuron type, calcium reporter, position of cut relative to the cell body, and the portion of cell analyzed. Strains expressing cameleon YC2.12 displayed a deficit in regeneration compared to non-cameleon strains at the 24 h time point (Figure S7). Although we observed a general reduction in overall regenerative outgrowth for all strains expressing the calcium-binding cameleon YC2.12, the ∼50% relative reduction in outgrowth compared to WT control is maintained in the *crt-1* mutant in the presence or absence of cameleon YC2.12, so basic conclusions on the requirement for *crt-1* are not compromised by the use of the cameleon YC2.12 reporter (Figure S7). The WT strain expressing cameleon YC3.60 showed no significant defect in regenerative outgrowth at the 5 h time point.

### Statistical Analysis

Details of statistical analysis are stated in the figure legends. In general, for comparisons between two measurements a two-tailed Student's *t* test was used to show statistical significance (direct *t* tests are indicated by brackets where they are not otherwise obvious). For group comparisons involving multiple strains (i.e., all strains within one figure panel unless otherwise indicated) the Dunn-Sidak group comparison method was used. Statistical tests were implemented using MATLAB (The MathWorks, Inc.). Outgrowth rates in Figure 2a insert were calculated by regression fits to the data as described above.

### *C. elegans* Strains and Media

Strains were grown at 20°C on NGM agar seeded with *Escherichia coli* OP50 as a food source [59]. The wild type strain was *C. elegans* N2 Bristol. Standard genetic techniques were used to generate compound mutant strains. The active site point mutation allele *ced-3(n2433)* was used in all compound mutant strains (see Table 1).

**Table 1:** List of strains used in this study.

Strain Name	Genotype
ZB2673	*zdIs5[p_mec-4_gfp]* I; *ced-3(n2433)* IV
ZB2676	*zdIs5[p_mec-4_gfp]* I; *ced-3(n2452)* IV
ZB2694	*zdIs5[p_mec-4_gfp]* I; *ced-3(n2888)* IV
ZB2677	*zdIs5[p_mec-4_gfp]* I; *ced-4(n1162)* III
ZB2699	*zdIs5[p_mec-4_gfp]* I; *ced-4(n1416)* III
ZB2678	*zdIs5[p_mec-4_gfp]* I; *ced-9(n1950)* III *gf*
ZB2675	*zdIs5[p_mec-4_gfp]* I; *ced-9(n2812)/qC1 dpy-19(e1259) glp-1(q339)* III; *ced-9(n2812) lf* homozygotes are viable due to maternal rescue, but sterile
ZB2674	*zdIs5[p_mec-4_gfp]* I; *egl-1(n1084n3082)* V
ZB2680	*zdIs5[p_mec-4_gfp]* I; *ced-8(n1891)* X
ZB2708	*lin-35(n745) zdIs5[p_mec-4_gfp]* I
ZB2689	*zdIs5[p_mec-4_gfp]* I; *ced-4(n1162)* III; *ced-3(n2433)* IV
ZB2701	*zdIs5[p_mec-4_gfp]* I; *egl-1(n1084n3082)* V; *ced-13(sv32)* X
ZB2698	*zdIs5[p_mec-4_gfp]* I; *egl-1(n986)* V
ZB2688	*oxIs12[p_unc-47_gfp]* X; *ced-3(n2433)* IV
ZB2679	*zdIs5[p_mec-4_gfp]* I; *crt-1(bz29)* V
ZB2700	*zdIs5[p_mec-4_gfp]* I; *crt-1(ok948)* V
ZB2684	*zdIs5[p_mec-4_gfp]* I; *ced-3(n2433)* IV; *crt-1(bz29)* V
ZB2705	*zdIs5[p_mec-4_gfp]* I; *crt-1(ok948)* V; *bzIs122[pmec-4ced-3 unc-119(+)]*
ZB2710	*crt-1(bz29)* V; *bzIs17[mec-4pYC2.12+lin-15 plasmid]*
ZB2711	*crt-1(ok948)* V; *bzIs17[mec-4pYC2.12+lin-15 plasmid]*
ZB2687	*zdIs5[p_mec-4_gfp]* I; *bzIs123[unc-119(+)]*
ZB2695	*zdIs5[p_mec-4_gfp]* I; *bzIs122[p_mec-4_ced-3]*
ZB2686	*zdIs5[p_mec-4_gfp]* I; *ced-3(n2433)* IV; *bzIs123[unc-119(+)]*
ZB2685	*zdIs5[p_mec-4_gfp]* I; *ced-3(n2433)* IV; *bzIs122[p_mec-4_ced-3]*
ZB2707	*dlk-1(ju476) zdIs5[p_mec-4_gfp]* I
ZB2709	*dlk-1(ju476) zdIs5[p_mec-4_gfp]* I; *ced-3(n2433)* IV
ZB4005	*dlk-1(ju476) zdIs5[p_mec-4_gfp]* I; *bzIs122[p_mec-4_ced-3]*
ZB4004	*zdIs5[p_mec-4_gfp]* I; *ced-4(n1162); bzIs122[p_mec-4_ced-3]*
ZB4006	*dlk-1(ju476) zdIs5[p_mec-4_gfp]* I; *crt-1(bz29)* V
ZB4007	*dlk-1(ju476) zdIs5[p_mec-4_gfp]* I; *ced-4(n1162)* III
ZB4010	*zdIs5[p_mec-4_gfp]* I; *kgb-1(um3)* IV
ZB4009	*zdIs5[p_mec-4_gfp]* I; *kgb-1(um3) ced-3(n2433)* IV
CG1B	*bzIs158[p_mec-4_YC3.6]*
ZB4008	*ced-3(n2433)* IV; *bzIs158[p_mec__-4_YC3.6]*
ZB4011	*ced-4(n1162)* III; *bzIs158[p_mec__-4_YC3.6]*
ZB154	*zdIs5[p_mec-4_gfp lin-15(+)]* I
EG1285	*oxIs12[p_unc-47_gfp lin-15(+)]* X
ZB1056	*lin-15(c11) X; bzIs17[p_mec-4_YC2.12 lin-15(+)]*
KWN177	*rnyIs014[p_mec-4_mCherry unc-119(+)]*

Mutations are loss-of-function unless otherwise indicated. We confirmed lesions in all *ced-3*, *ced-4*, and *dlk-1* constructs by DNA sequence analysis.

### Note on Molecular Lesions of Alleles Studied

The true *ced-3* null allele has not been formally defined, although many loss-of-function mutants have been described in detail [60]. All four *ced-3* alleles studied are strong loss-of-function. The *n2433* allele encodes a point mutation that alters the caspase active site and shows weak semi-dominance regarding apoptosis; the encoded substitution generates a mutant CED-3 that has no detectable protease activity in vitro [14]. We also studied regeneration in *ced-3(n2452)* (a 17 Kb deletion also disrupting four other putative genes: C48D1.1, F58D2.2, F58D2.4, and F58D2.1), *ced-3(n717)* (mutation of the conserved acceptor site of intron #7), and *ced-3(n2888)* (early stop codon).

The *crt-1(ok948)* deletion mutant deletes all but the first 21 amino acids, including the stop codon. *crt-1(bz29)* encodes a stop codon at position 28 and lacks immunoreactivity [61]. These *crt-1* alleles have been suggested to be functional null alleles.

The *dlk-1(ju476)* allele is a 5 bp insertion at G631 [62]; this allele has been cited to act as a null allele for axonal regeneration [9].

### Plasmid Construction and Generation of *ced-3* Transgenic Animals

Plasmids were constructed using standard genetic techniques. The *p_mec-4_mCherry* vector was constructed by amplification of mCherry sequence improved for expression in *C. elegans* [63] using the following primers: 5′-GGGATCCATGGTCTCAAAGGGTGAAGA-3′ and 5′-GGAATTCTTATACAATTCATCCATGCC-3′. The PCR fragment generated was cloned into *p_mec-4_GFP* [64], replacing GFP using *Bam*HI and *Eco*RI sites.

For the construction of *p_mec-4_ced-3*, *ced-3* cDNA was amplified from a pool of *C. elegans* cDNA using primers 5′-GGATCCATGATGCGTCAAGATAGAAGGA-3′ and 5′-CAATTGTTAGACGGCAGAGTTTCGTGC-3′ and cloned into pCR2.1 using TOPO TA cloning kit (Invitrogen). For further cloning purposes, the *Hind*III site of *ced-3* cDNA was inactivated while introducing the silent mutation A to G at position 609 on the cDNA giving pCR*ced-3(A609G)* (QuikChange II Site-Directed Mutagenesis Kit). The GFP fragment of *p_mec-4_GFP* [64] was replaced with *ced-3(A609G)* from pCR*ced-3(A609G)* using *BamH*I and *Mfe*I sites. A fragment containing the *mec-4* promoter fused to *ced-3(A609G)* cDNA from the previous vector was introduced using *Apa*I and *Hind*III sites into pDP#MM016b bearing *unc-119(+)* [65] and giving the *p_mec-4_ced-3* vector construction.

The *p_mec-4_ced-3* vector and the pDP#MM016b [65] vector bearing *unc-119* gene were used for bombarding *unc-119(ed3)* animals as described [66]. Generated transgenic lines were *bzIs122[p_mec-4_ced-3 unc-119(+)]* and *bzIs123[unc-119(+)]*, named *Is[p_mec-4_ced-3]* and *Is[unc-119(+)]*, respectively, in the figures presented for this study. Strains were outcrossed once before further genetic constructions. Note that the line generated exhibited evidence of some touch neuron loss (Figure S2) and that numerous repeated attempts at generation of transgenic expression of *C. elegans* caspase genes were unsuccessful. This is likely due to the toxicity of elevated *ced-3* expression. Note that although we obtained published lines overexpressing *dlk-1* on extrachromosomal arrays, transgenic lines were consistently sick and array transgenes were lost at a very high frequency, precluding our ability to test *dlk-1* overexpression in *ced-3* mutants.

## Supporting Information

Figure S1 The severed distal fragment generated consequent to ALM axotomy often persists for days. (a) Pictures of a regenerating ALM neuron expressing the *zdIs5[pmec-4gfp]* transgene that does not obviously regrow to the site of the dissociated fragment. Note that the severed distal end (green arrow), disconnected from the cell body, remains visible for at least 3 d post-axotomy in young adults. Red arrow indicates laser cut point. (b) To quantitate process persistence in non-reconnected neurons, we classified degeneration of the distal fragment into three types: (i) no or very minimal degeneration (apart from the formation of an end bulb at the cut point); (ii) significant degeneration consisting of apparent thinning of the axon, significant loss of GFP fluorescence, and/or beading; and (iii) fragmentation and complete degeneration (this was not observed). (c) Degeneration from ALM axotomies classified in this way in wild type (*N* = 39) and *ced-3(n2433)* mutant (*N* = 37) animals showed no significant difference.
(TIF)

Figure S2 CED-3 caspase expression affects neuronal health, but one minimally toxic low copy number line can be used for rescue in touch receptor neurons. To test whether *ced-3*-specific expression in touch neurons could rescue the *ced-3* mutation defect in regeneration, we constructed transgenic lines using biolistic transformation, which generates low copy number integrated transgenes. We first engineered a control transgenic line harboring an integrated *unc-119* gene (the selectable marker used for biolistic transformation, which is also a critical gene for neuronal development, see details in Materials and Methods), indicated as *Is[unc-119(+)]*. *Is[unc-119(+)]* was crossed to the *ced-3(n2433)* mutant to generate *ced-3(n2433); Is[unc-119(+)].* The *p_mec-4_ced-3* cDNA was expressed in touch neurons in the wild type and *ced-3(n2433)* backgrounds along with co-transformation marker *unc-119*. These strains are indicated as *Is[p_mec-4_ced-3]* and *ced-3(n2433); Is[p_mec-4_ced-3]*. To test for toxicity associated with *ced-3* neuronal expression from *Is[p_mec-4_ced-3]*, we compared surviving fluorescent touch neurons visualized by the *zdIs5[p_mec-4_gfp*]transgene in wild type, *ced-3(n2433)*, the control transgenic strains *Is[unc-119(+)]* and *ced-3(n2433); Is[unc-119(+)]*, as well as transgenic strains expressing *ced-3* in the touch neurons *Is[p_mec-4_ced-3]* and *ced-3(n2433); Is[p_mec-4_ced-3]*. Mean ± s.e.m. are shown. Student's *t* test, with a Dunn-Sidak adjustment for multiple comparisons, was used to determine the statistical significance: *p<0.005 versus wild type, ^#^ *p*<0.005 versus *ced-3(n2433).* Note that *ced-3* mutant displays 1.1±0.8 (mean ± standard deviation) extra surviving fluorescent neurons as compared to wild type, including in transgenic backgrounds, consistent with a previous report suggesting survival of a lineage sister that does not undergo programmed cell death in this background [67]. Many transgene lines had higher levels of touch neuron death associated with *ced-3* overexpression (not shown) and thus could not be used for rescue assays in our study.
(TIF)

Figure S3 Immobilized *C. elegans* in microfluidics channels. *C. elegans* were physically immobilized in microfluidic devices consisting of a parallel array of 128 tapered channels or worm “clamps.” Constant suction through the device sufficiently restrained the animals for laser surgery and subsequent time-lapse imaging. This figure is related to time lapse imaging quantitated in Figure 2a.
(TIF)

Figure S4 Growth cones of *ced-3* mutant neurons exhibit wild type behavior during development. Migrating VD neurons exhibit stereotyped behaviors when they contact a new substratum, as visualized by the *oxIs12[unc-47::GFP]* in the wild type and in *ced-3(n2433)* mutant. Rounded growth cones migrate across the epidermis (left panels). Growth cones form anvils at the lateral nerve cord (middle panels). Anvil-shaped growth cones paused at the dorsal body wall muscle extend fingers toward the dorsal nerve cord (right panels). Five larvae were observed for each genotype. Pictures are projections of z-stacks. The scale bar represents 5 µm. We conclude that *ced-3* mutants do not have major systemic defects in developmental growth cones.
(TIF)

Figure S5 Post-axotomy regenerative dynamics in the dissociated distal axon segment reveal that CED-3 activities can be induced in a cellular fragment devoid of a nucleus. (a) Mean time of initial outgrowth from the severed end of the distal fragment after laser surgery for WT (grey) and *ced-3(n2433)* (red) mutant (see Figure 2). (b) Mean number of individual exploratory processes generated from the dissociated end of the distal axon segment, during the 0–5 h time period following laser surgery. Student's *t* test was used to determine the statistical significance of differences for *ced-3* versus WT in each panel; *p<0.05. See also Movies S1, S2, and S3 for views of changes in dissociated distal ends.
(TIF)

Figure S6 Regeneration efficiency is lower in *kgb-1 ced-3* than in *ced-3* and *kgb-1* strains, suggesting that *kgb-1* and *ced-3* act in different regeneration pathways. A recent study suggested parallel kinase pathways promote *C. elegans* regeneration, and that *kgb-1* was one kinase that might act in parallel to *dlk-1* [18]. Because our genetic data suggested that *dlk-1* acts in the *ced-3* pathway, we elected to construct a double mutant with *kgb-1* to provide proof-of-principle that double mutants impacting parallel pathways would have enhanced regeneration defects. We measured regenerative outgrowth of the axotomized ALM neuron visualized using the *zdIs5[pmec-4gfp]* transgene and monitored 24 h post-surgery in *ced-3(n2433)* and *kgb-1(um3)* single mutants and in the *kgb-1(um3) ced-3(n2433)* double mutant. Student's *t* test, with a Dunn-Sidak adjustment for multiple comparisons, was used to determine the statistical significance: **p<0.05 versus WT, #*p*<0.05 versus *kgb-1(um3) ced-3(n2433).*
(TIF)

Figure S7 *crt-1* mutant axons exhibit reduced regenerative outgrowth with calcium sensor cameleon YC2.12 in the background. Since calcium-binding cameleon might sequester calcium to change regeneration events when expressed in touch neurons, we scored our cameleon strains for regenerative outgrowth. Both WT and *crt-1* strains that harbor cameleon YC2.12 transgenes exhibit diminished regenerative outgrowth as compared to non-cameleon strains (WT shown, compare *crt-1* data with Figure 6b). However, even with cameleon transgene expression, *crt-1* mutants remain ∼50% reduced in 24 h regenerative outgrowth such that conclusions on calcium signaling remain valid (see Figure 6a). The wild type strain expressing improved cameleon variant YC3.60 showed no significant defect in regenerative outgrowth at the 5 h time point but was not investigated further since a *crt-1* dependence for efficient regeneration was apparent even with YC2.12. Shown is mean regenerative outgrowth 24 h after laser surgery for strains expressing the cameleon YC2.12 *bzIs17[p_mec-4_YC2.12+lin-15(+)]* (indicated as WT YC2.12), *bzIs17[p_mec-4_YC2.12+lin-15(+)]; crt-1(bz29)* (indicated as *crt-1(bz29)* YC2.12), and *bzIs17[p_mec-4_YC2.12+lin-15(+)]; crt-1(ok948)* (indicated as *crt-1(ok948)* YC2.12). Brackets represent Student's *t* test between the two indicated measurements, with *p<0.05, **p<0.005.
(TIF)

Movie S1 Regeneration of an ALM neuron after femtosecond laser axotomy. The ALM dendrite was targeted 13 µm from the cell body in an adult wild type *C. elegans* (arrow). We can visualize new growth cones that direct axon extension. Note that the posterior process also initiates limited outgrowth. Frames were taken every 15 min as indicated with laser axotomy occurring at T = 0 min. Duration, 9 h 30 min. Scale bar, 10 µm.
(AVI)

Movie S2 Time-lapse regenerative dynamics in WT. Representative time-lapse movies of initial stages (0–5 h) of neuronal regeneration. ALM neurons display numerous transient, dynamically active exploratory processes. Animals were held in microfluidic devices for laser surgery and time-lapse imaging (see Materials and Methods and Figure S3). Frames were taken every 10 min as indicated with laser axotomy occurring at T = 0 min. Scale bar, 10 µm. Select frames are displayed in Figure 2d.
(AVI)

Movie S3 Time-lapse regenerative dynamics in *ced-3* mutant. Representative time-lapse movies of initial stages (0–5 h) of neuronal regeneration in *ced-3(n2433)* mutant background. *ced-3* mutants display significantly fewer of these extensions, and initial outgrowth is substantially delayed as compared to WT. Animals were held in microfluidic devices for laser surgery and time-lapse imaging (see Materials and Methods and Figure S3). Frames were taken every 10 min as indicated with laser axotomy occurring at T = 0 min. Scale bar, 10 µm. Select frames are displayed in Figure 2e.
(AVI)

## Abbreviations

CNS: central nervous system
*gf*: gain-of-function
*lf*: loss-of-function
VNC: ventral nerve cord
WT: wild type
