# Supplementary figures and images for: The Core Apoptotic Executioner Proteins CED-3 and CED-4 Promote Initiation of Neuronal Regeneration in Caenorhabditis elegans
Source: PLoS Biol. 2012 May 22;10(5):e1001331. doi: 10.1371/journal.pbio.1001331 (PMC3358320; doi:10.1371/journal.pbio.1001331)

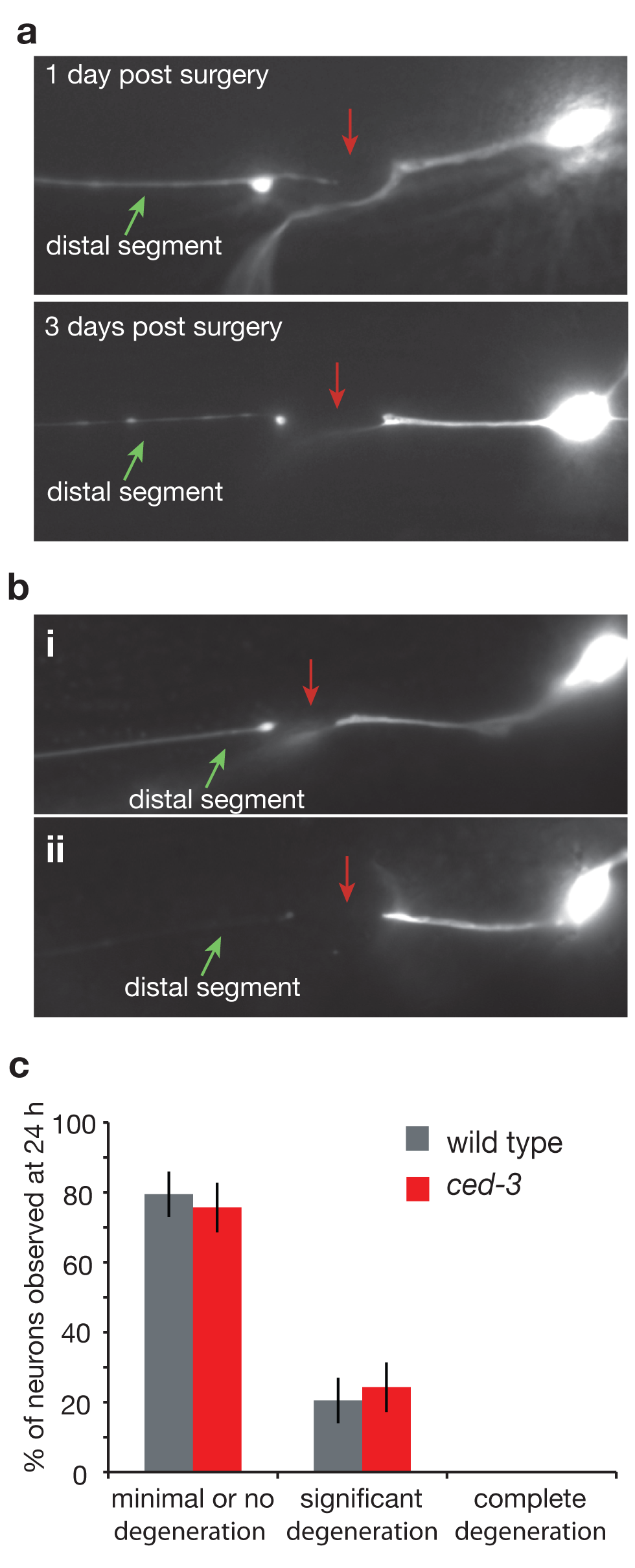

Supplement: Figure S1 — The severed distal fragment generated consequent to ALM axotomy often persists for days. (a) Pictures of a regenerating ALM neuron expressing the zdIs5[pmec-4gfp] transgene that does not obviously regrow to the site of the dissociated fragment. Note that the severed distal end (green arrow), disconnected from the cell body, remains visible for at least 3 d post-axotomy in young adults. Red arrow indicates laser cut point. (b) To quantitate process persistence in non-reconnected neurons, we classified degeneration of the distal fragment into three types: (i) no or very minimal degeneration (apart from the formation of an end bulb at the cut point); (ii) significant degeneration consisting of apparent thinning of the axon, significant loss of GFP fluorescence, and/or beading; and (iii) fragmentation and complete degeneration (this was not observed). (c) Degeneration from ALM axotomies classified in this way in wild type (N = 39) and ced-3(n2433) mutant (N = 37) animals showed no significant difference. (TIF) [file pbio.1001331.s001.tif]

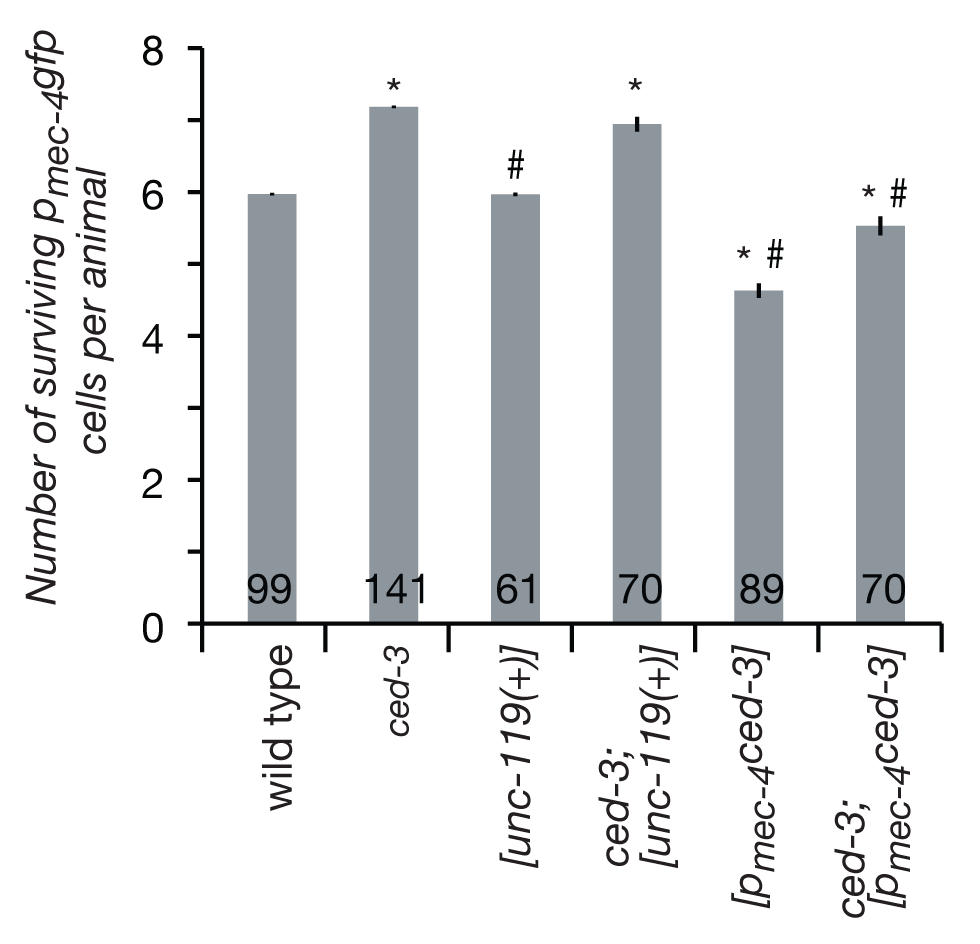

Supplement: Figure S2 — CED-3 caspase expression affects neuronal health, but one minimally toxic low copy number line can be used for rescue in touch receptor neurons. To test whether ced-3-specific expression in touch neurons could rescue the ced-3 mutation defect in regeneration, we constructed transgenic lines using biolistic transformation, which generates low copy number integrated transgenes. We first engineered a control transgenic line harboring an integrated unc-119 gene (the selectable marker used for biolistic transformation, which is also a critical gene for neuronal development, see details in Materials and Methods), indicated as Is[unc-119(+)]. Is[unc-119(+)] was crossed to the ced-3(n2433) mutant to generate ced-3(n2433); Is[unc-119(+)]. The pmec-4ced-3 cDNA was expressed in touch neurons in the wild type and ced-3(n2433) backgrounds along with co-transformation marker unc-119. These strains are indicated as Is[pmec-4ced-3] and ced-3(n2433); Is[pmec-4ced-3]. To test for toxicity associated with ced-3 neuronal expression from Is[pmec-4ced-3], we compared surviving fluorescent touch neurons visualized by the zdIs5[pmec-4gfp]transgene in wild type, ced-3(n2433), the control transgenic strains Is[unc-119(+)] and ced-3(n2433); Is[unc-119(+)], as well as transgenic strains expressing ced-3 in the touch neurons Is[pmec-4ced-3] and ced-3(n2433); Is[pmec-4ced-3]. Mean ± s.e.m. are shown. Student's t test, with a Dunn-Sidak adjustment for multiple comparisons, was used to determine the statistical significance: *p<0.005 versus wild type, # p<0.005 versus ced-3(n2433). Note that ced-3 mutant displays 1.1±0.8 (mean ± standard deviation) extra surviving fluorescent neurons as compared to wild type, including in transgenic backgrounds, consistent with a previous report suggesting survival of a lineage sister that does not undergo programmed cell death in this background [67]. Many transgene lines had higher levels of touch neuron death associated with ced-3 overexpression (not shown) and [file pbio.1001331.s002.tif]

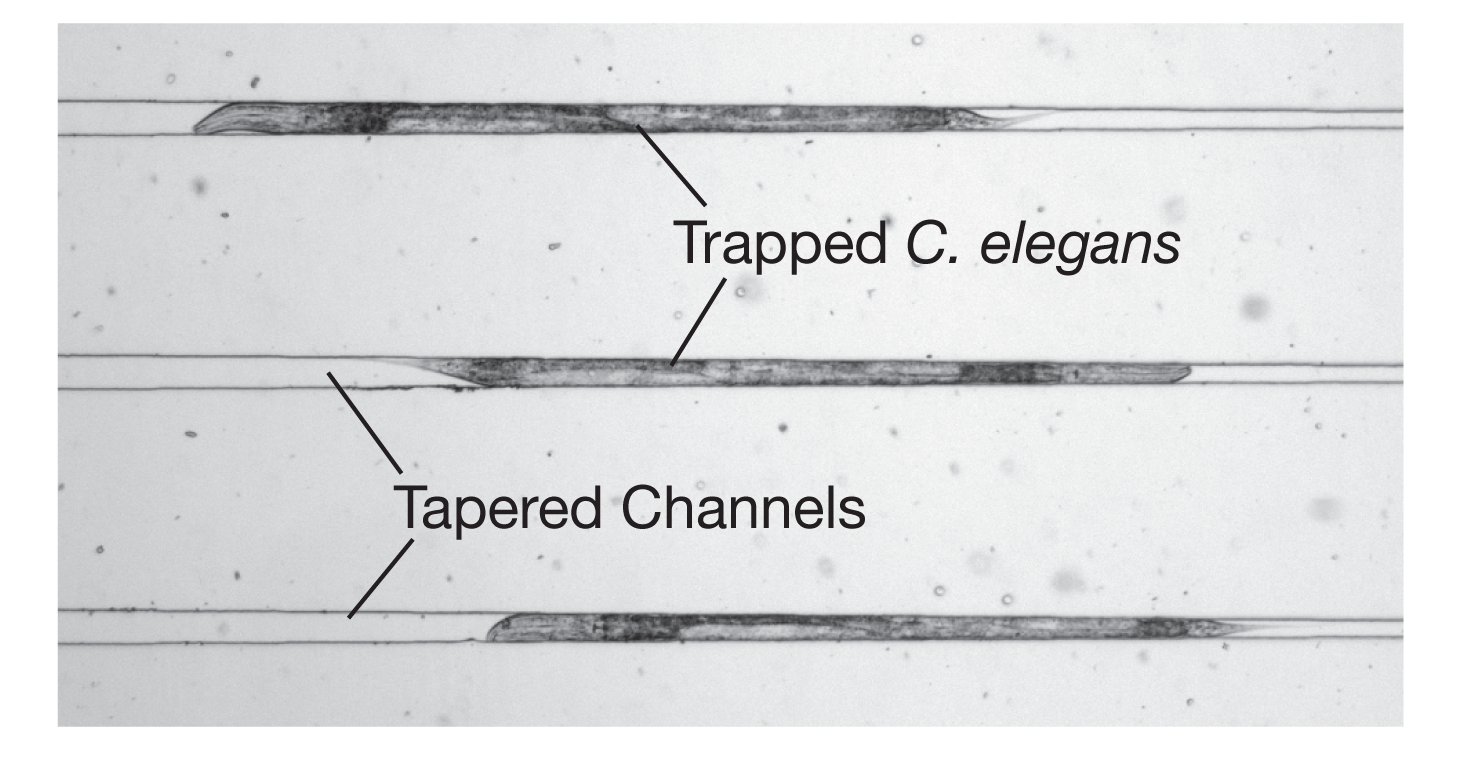

Supplement: Figure S3 — Immobilized C. elegans in microfluidics channels. C. elegans were physically immobilized in microfluidic devices consisting of a parallel array of 128 tapered channels or worm “clamps.” Constant suction through the device sufficiently restrained the animals for laser surgery and subsequent time-lapse imaging. This figure is related to time lapse imaging quantitated in Figure 2a. (TIF) [file pbio.1001331.s003.tif]

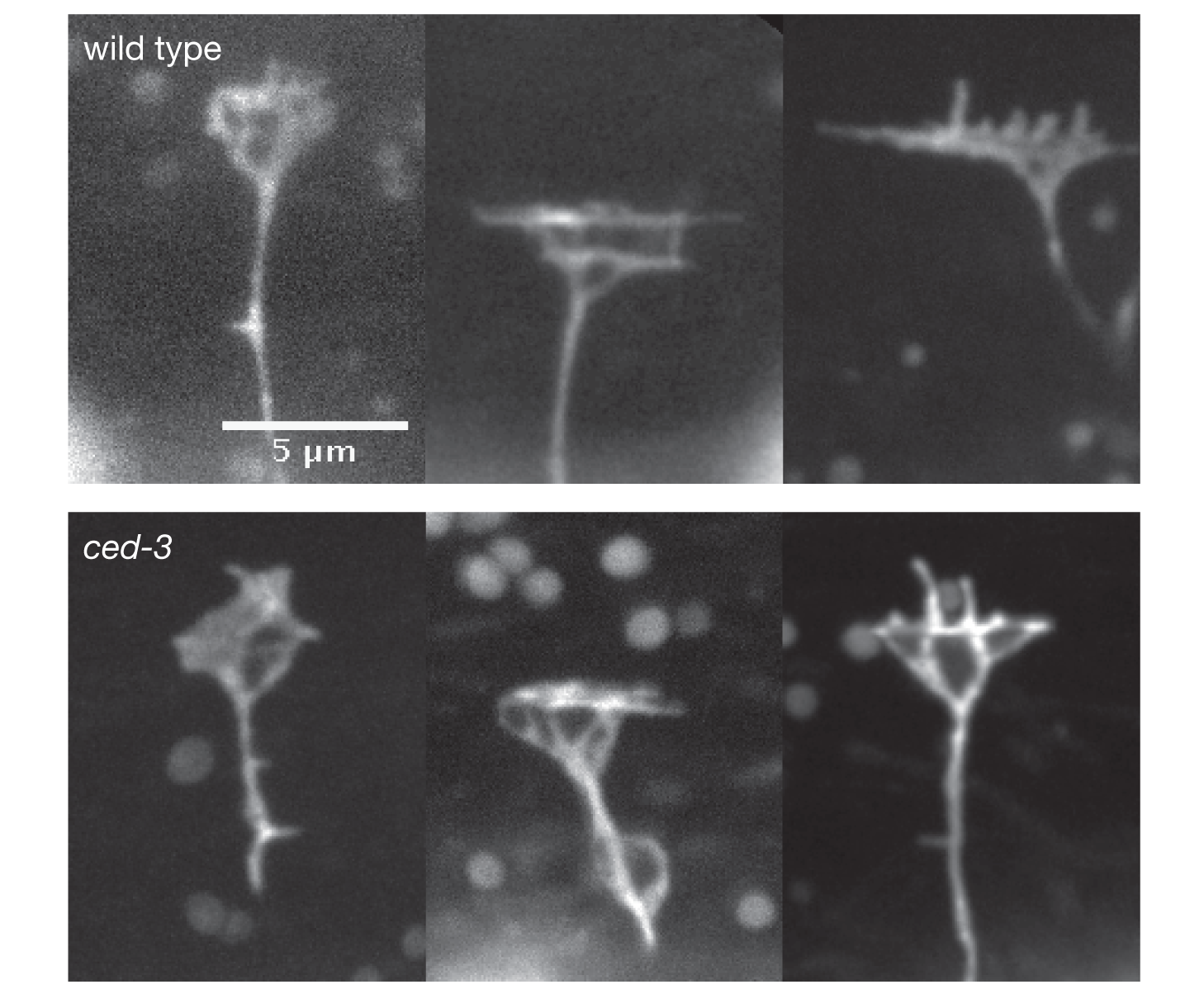

Supplement: Figure S4 — Growth cones of ced-3 mutant neurons exhibit wild type behavior during development. Migrating VD neurons exhibit stereotyped behaviors when they contact a new substratum, as visualized by the oxIs12[unc-47::GFP] in the wild type and in ced-3(n2433) mutant. Rounded growth cones migrate across the epidermis (left panels). Growth cones form anvils at the lateral nerve cord (middle panels). Anvil-shaped growth cones paused at the dorsal body wall muscle extend fingers toward the dorsal nerve cord (right panels). Five larvae were observed for each genotype. Pictures are projections of z-stacks. The scale bar represents 5 µm. We conclude that ced-3 mutants do not have major systemic defects in developmental growth cones. (TIF) [file pbio.1001331.s004.tif]

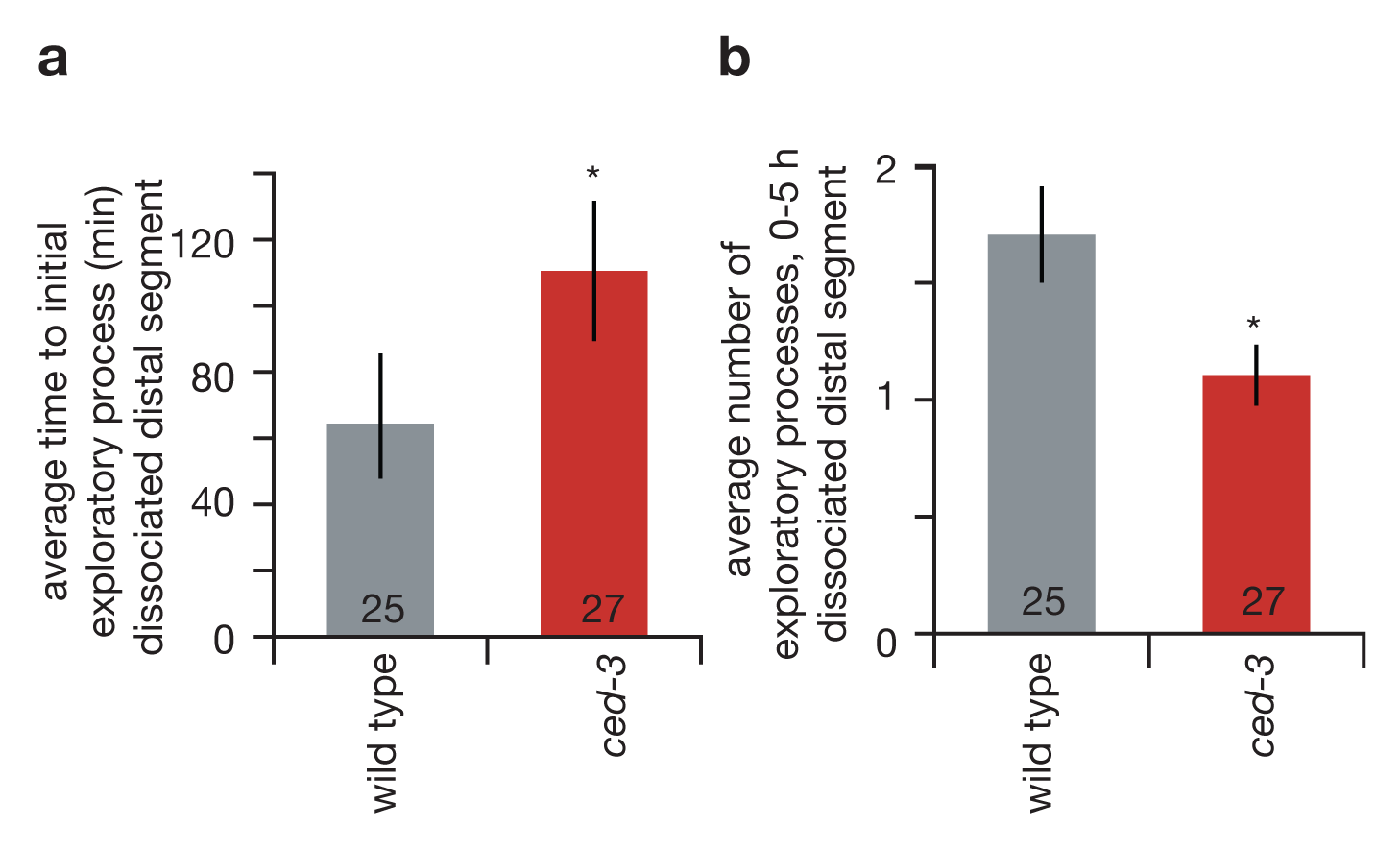

Supplement: Figure S5 — Post-axotomy regenerative dynamics in the dissociated distal axon segment reveal that CED-3 activities can be induced in a cellular fragment devoid of a nucleus. (a) Mean time of initial outgrowth from the severed end of the distal fragment after laser surgery for WT (grey) and ced-3(n2433) (red) mutant (see Figure 2). (b) Mean number of individual exploratory processes generated from the dissociated end of the distal axon segment, during the 0–5 h time period following laser surgery. Student's t test was used to determine the statistical significance of differences for ced-3 versus WT in each panel; *p<0.05. See also Movies S1, S2, and S3 for views of changes in dissociated distal ends. (TIF) [file pbio.1001331.s005.tif]

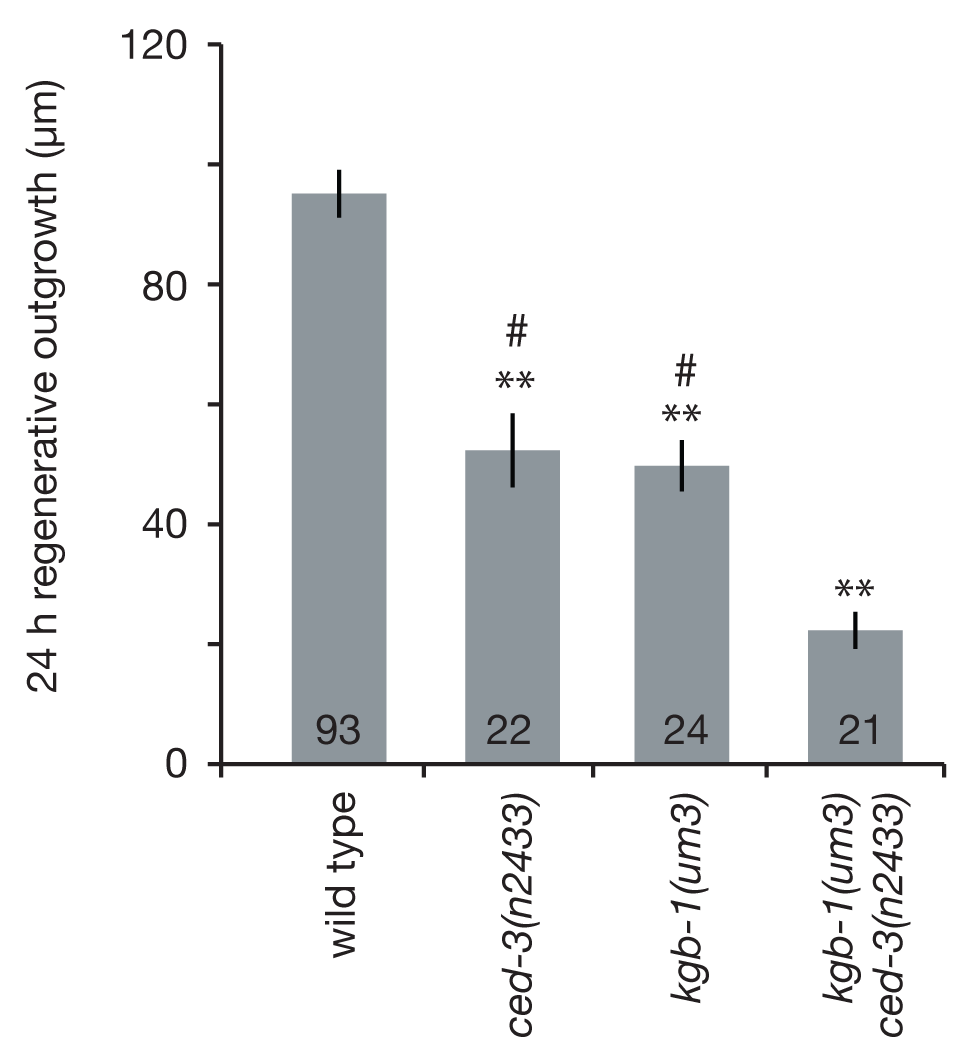

Supplement: Figure S6 — Regeneration efficiency is lower in kgb-1 ced-3 than in ced-3 and kgb-1 strains, suggesting that kgb-1 and ced-3 act in different regeneration pathways. A recent study suggested parallel kinase pathways promote C. elegans regeneration, and that kgb-1 was one kinase that might act in parallel to dlk-1 [18]. Because our genetic data suggested that dlk-1 acts in the ced-3 pathway, we elected to construct a double mutant with kgb-1 to provide proof-of-principle that double mutants impacting parallel pathways would have enhanced regeneration defects. We measured regenerative outgrowth of the axotomized ALM neuron visualized using the zdIs5[pmec-4gfp] transgene and monitored 24 h post-surgery in ced-3(n2433) and kgb-1(um3) single mutants and in the kgb-1(um3) ced-3(n2433) double mutant. Student's t test, with a Dunn-Sidak adjustment for multiple comparisons, was used to determine the statistical significance: **p<0.05 versus WT, #p<0.05 versus kgb-1(um3) ced-3(n2433). (TIF) [file pbio.1001331.s006.tif]

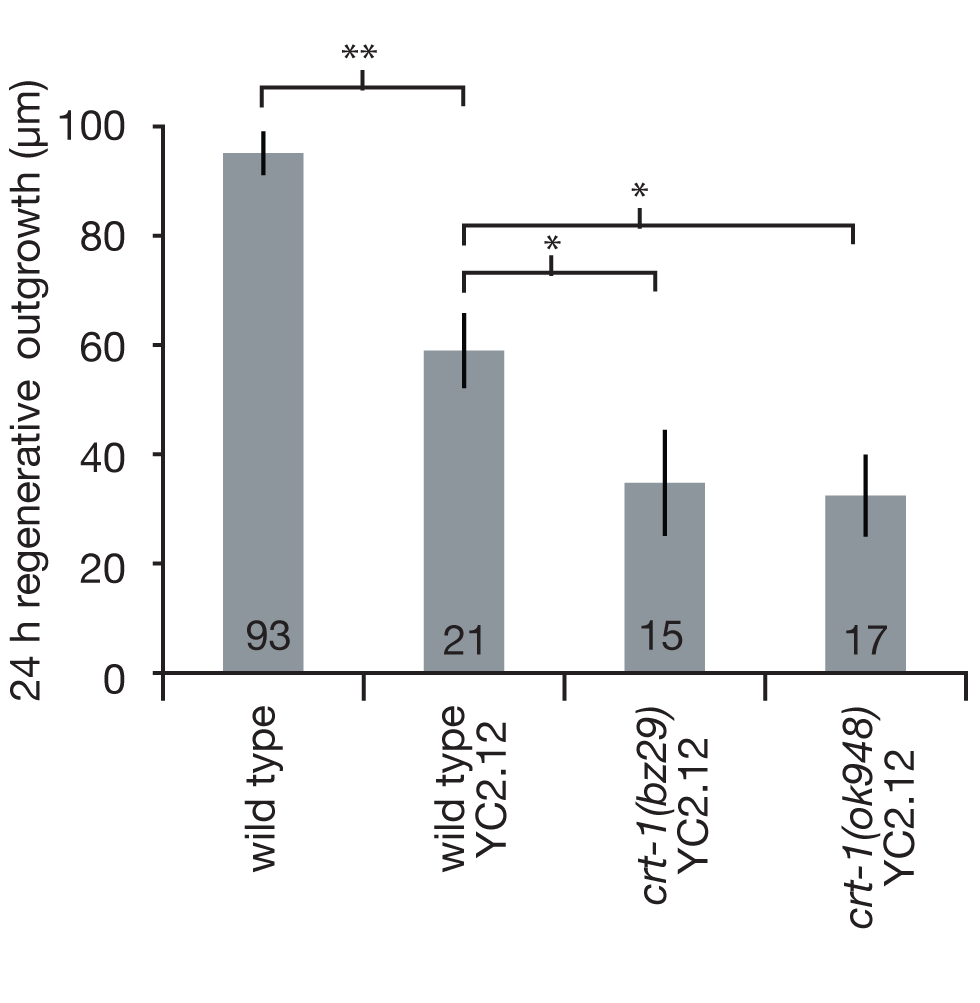

Supplement: Figure S7 — crt-1 mutant axons exhibit reduced regenerative outgrowth with calcium sensor cameleon YC2.12 in the background. Since calcium-binding cameleon might sequester calcium to change regeneration events when expressed in touch neurons, we scored our cameleon strains for regenerative outgrowth. Both WT and crt-1 strains that harbor cameleon YC2.12 transgenes exhibit diminished regenerative outgrowth as compared to non-cameleon strains (WT shown, compare crt-1 data with Figure 6b). However, even with cameleon transgene expression, crt-1 mutants remain ∼50% reduced in 24 h regenerative outgrowth such that conclusions on calcium signaling remain valid (see Figure 6a). The wild type strain expressing improved cameleon variant YC3.60 showed no significant defect in regenerative outgrowth at the 5 h time point but was not investigated further since a crt-1 dependence for efficient regeneration was apparent even with YC2.12. Shown is mean regenerative outgrowth 24 h after laser surgery for strains expressing the cameleon YC2.12 bzIs17[pmec-4YC2.12+lin-15(+)] (indicated as WT YC2.12), bzIs17[pmec-4YC2.12+lin-15(+)]; crt-1(bz29) (indicated as crt-1(bz29) YC2.12), and bzIs17[pmec-4YC2.12+lin-15(+)]; crt-1(ok948) (indicated as crt-1(ok948) YC2.12). Brackets represent Student's t test between the two indicated measurements, with *p<0.05, **p<0.005. (TIF) [file pbio.1001331.s007.tif]
